# Engineered exosomes derived from miR-132-overexpresssing adipose stem cells promoted diabetic wound healing and skin reconstruction

**DOI:** 10.3389/fbioe.2023.1129538

**Published:** 2023-03-01

**Authors:** Lifeng Ge, Kangyan Wang, Hang Lin, Endong Tao, Weijie Xia, Fulin Wang, Cong Mao, Yongzeng Feng

**Affiliations:** ^1^ Department of Orthopaedics, The Second Affiliated Hospital and Yuying Children’s Hospital of Wenzhou Medical University, Wenzhou, China; ^2^ Zhejiang Provincial Key Laboratory of Orthopaedics, Wenzhou Medical University, Wenzhou, China

**Keywords:** diabetic wound, miR-132, exosomes, macrophages polarization, inflammation

## Abstract

The tissue reconstruction of diabetic wounds mainly depends on the proliferation and remodelling of cutaneous cells around wounds and the transplantation of random skin flaps, however, the proliferation of cells or survival of skin flaps are difficult due to the severe inflammation and other problems caused by diabetes. The stem cell-derived exosomes loaded with miRNA can be an effective therapeutic strategy for promoting diabetic wound healing. Therefore, in this study, the engineered exosomes derived from miR-132-overexpressing adipose stem cells (miR-132-exo) was obtained for promoting the healing of diabetic wounds and skin flaps. *In vitro*, the miR-132-exo promoted the proliferation and migration of human umbilical vein endothelial cells (HUVECs). *In vivo*, streptozotocin (STZ) induced diabetic mice were used to create full-thickness skin wounds and random skin flaps to further investigate the healing effect of miR-132-exo. The results showed miR-132-exo evidently enhanced the survival of skin flaps and promote diabetic wound healing, through reducing local inflammation, promoting angiogenesis and stimulating M2-macrophages polarization mediated by NF-κB signaling pathway. These novel findings demonstrated that engineered miR-132-exo can be a potent therapeutic for treating diabetic wounds and inflammatory-related disease.

## 1 Introduction

The repair of diabetic wounds remains a major concern in clinic (Zhang et al., 2022). The healing of skin wound is mainly depending on the proliferation and migration of several kinds of cells surrounding wounds and/or the survival of transplanted skin random flaps (Park and Park, 2022). However, due to diabetes, patients usually suffer from metabolic disorders, impaired immune functions, and slow nerve activity that causes excessive inflammation, cellular dysfunction, and other problems (Thomas et al., 2020). On one hand, excessive and continuous inflammation inhibits cell proliferation and migration that is necessary for wound repair; on the other hand, the dysfunction of vascular endothelial cells and microcirculation disorders causes the difficulty of vascularization of the wounds or the transplanted skin flaps (Vu et al., 2021). In that case, these grafted skin flaps are at high risk of ischemia and necrosis (Mao et al., 2019), and it’s difficult to regenerate skin tissue due to cell dysfunction and inadequate blood supply. Therefore, diabetic wounds are often difficult to heal on their own.

In recent years, many studies have shown the stem cells may exert their functions through the paracrine mechanism, particularly through releasing extracellular vesicles to regulate the function and signal of target cells (Gentile and Garcovich, 2019). Exosomes (Exo) secreted by various cells are active vesicles with a size of about 40–150 nm (Kalluri and LeBleu, 2020). Exosomes are rich in bioactive substances, including mRNAs, miRNAs, and proteins, which can be received by target cells through the process of endocytosis (Hu et al., 2020), which also shows the importance of exosomes for cell-to-cell communication (Pitt et al., 2016). Previous studies have shown that exosomes derived from adipose stem cells can promote the healing process through accelerating cell proliferation and tissue vascularization in wounds (Li et al., 2018). Another exciting point of exosomes is that due to their physicochemical stability *in vivo* and the characteristics of multidimensional packaging, the miRNA loaded by exosomes is not easy to be degraded and can be modified and customized by primitive cells (Desdín-Micó and Mittelbrunn, 2017). Therefore, the effective decoration of exosomes derived from ADSCs, such as the loading the functional miRNA, has a good prospect for diabetic wound healing.

Wound repair is a dynamic and complex process that mainly includes inflammation, proliferation, and remodeling phases (Wilkinson and Hardman, 2020). The inflammatory environment of the normal wound can help resist the invasion of bacteria, and can secrete cytokines that are conducive to wound repair. However, diabetic wound inflammation can cause more severe inflammation and further damage the wound (Li et al., 2015; Davis et al., 2018; Hodge et al., 2022a; Hodge et al., 2022b). Li et al. reported that the level of miR-132 was dramatically decreased in diabetic wounds and the local use of miR-132 can accelerate the diabetic wound healing by alleviating the inflammation through NF-κB signaling pathways (Li et al., 2017; Liu et al., 2017). Another study by Essandoh et al. found that miR-132 can switch M1 macrophages polarization into M2 state by targeting multiple transcription factors and adaptor proteins (Essandoh et al., 2016), which can be beneficial for anti-inflammation and further healing. The hyperglycemic environment of diabetic patients impairs the conversion of macrophages from a M1 pro-inflammatory state to an M2 anti-inflammatory state and leads to hyperinflammation and vascular circulation problems in diabetic wounds (Du et al., 2018; Aitcheson et al., 2021). In addition, miR-132 is a specific pro-angiogenic miRNA (Cannavicci et al., 2022) that has been proved to enhance angiogenesis in ischemic related diseases (Che et al., 2018; Ma et al., 2018). Shruti (Rawal et al., 2017) et al. also performed function loss experiments by using anti-miR-132 to inhibit the activity of miR-132 in HUVECs cultured with high glucose and discovered that downregulation of miR-132 was the leading cause of vascular endothelial dysfunction. Hence, the use of miR-132 can be effective for diabetic wound healing due to its anti-inflammation ability and angiogenic effect. However, bare microRNAs are easily hydrolyzed in wound micro-environment, and only a small number of microRNAs can be endocytosed by tissue cells to play a role in actual treatment process (Qian et al., 2020). Therefore, we use an engineering method to effectively obtain exosomes overexpressing miR-132 for promoting diabetic wound healing, which can protect miR-132 from degradation and increase the intracellular uptake of miR-132 to target cells (Lv et al., 2020).

In this study, engineered exosomes derived from miR-132-overexpressing adipose stem cells (miR-132-exo) were obtained through lentiviral transduction and ultracentrifuge method for promoting diabetic wound healing. *In vitro*, the effect of miR-132-exo on the proliferation and migration of HUVECs was investigated. *In vivo*, a diabetic wound model and a diabetic random skin flap model were established to study the healing effect of miR-132-exo. The former animal model mimicked the hyperactivated and persistent chronic inflammatory environment in diabetic wound remodelling. The latter simulated the situation of the impairment of endothelial function and microcirculation caused in diabetes mellitus, which leads to the difficulty of revascularization after flap transplantation. Further, the related mechanism about miR-132-exo inducing M2 macrophage polarization was studied. The results of this study will provide a new direction for the application of ADSC-exo as bioactive carriers by engineering miRNAs or other molecules into exosomes, and also provide new strategies for the clinical treatment of diabetic wound healing and improving the survival rate of skin flap.

## 2 Materials and methods

### 2.1 Cell culture and lentivirus transduction of adipose stem cells

The mouse adipose mesenchymal stem cells used in this study were purchased from OriCell^®^ (MUBMD-01001) and cultivated in Dulbecco’s modified Eagle’s medium (DMEM) with 10% fetal bovine serum (FBS) and low glucose. The lentivirus used in this experiment was constructed by Shanghai Jikai Technology Co., LTD. The lentivirus carrying the green fluorescent protein (GFP) tag of murine miR-132 was named as LV-MMU-miR-132. LV-MMU-miR-132 was then transfected into ADSCs to obtain ADSCs overexpressing miR-132 (miR-132-ADSCs), and untreated ADSCs were used as control. For transduction, ADSCs were cultured in medium (DMEM) and incubated with lentivirus for 24 h at a multiplicity of infection (MOI) of 90%. After that, the medium was renewed, and these ADSCs will be used for the following experiments.

### 2.2 Extraction and characterization of exosomes

The cells were spread in medium (DMEM) with 10% FBS and cultured to a density of approximately 70%. The medium was then replaced by 5% exosome-depleted FBS, and ADSCs were cultured for another 48 h. The medium was then collected and centrifuged at 4°C at 1,500 g for 10 min, followed by another 10 min at 2000 g, to remove residual cell debris. The supernatant was transferred to a centrifuge tube and centrifuged at 4°C at 10,000 g for 30 min. The supernatant obtained from the above process was transferred to a sterile centrifuge tube of the same specification, and then centrifuged at 4°C at 100,000 g for 70 min. The supernatant was removed and the obtained precipitation was re-suspended with PBS. In order to further purify exosomes, the suspension was filtered by a 0.22 μm filter membrane, then centrifuged at 4°C at 100,000 g for 60 min. After removing the supernatant, the exosomes were obtained and suspended in 100 μL PBS for further use. Transmission electron microscopy (TEM) (JEM-1400) was used to analyze the ultrastructure and morphology of exosomes, while nanoparticle tracking analysis was used to evaluate size distribution and nanoparticle concentration (NTA, PMX 110, particle matrix). The concentration of the extracted exosome solution was determined by a BCA kit and then diluted to a uniform concentration using the special solvent in the kit. Proteins from exosomes were extracted using an Exosome Protein extraction kit (Invitrogen) according to the manufacturer’s instructions, and the rest of exosomes were stored at −80°C for following experiments.

### 2.3 Quantitative real-time polymerase chain reaction

The expression levels of miR-132 in miR-132-exo and exo were evaluated by the RT-qPCR. Briefly, total RNA was extracted from exosome samples using RNA Extraction (Servicebio) and quantified by Nanodrop 2000 (Thermo Scientific). Then mRNA was then reverse transcribed into cDNA by SweScript RT I First Strand cDNA Synthesis Kit (Servicebio G3330) for miR-132 expression analysis, and cDNA amplification was performed using a Real-Time PCR System (Bio-Rad CFX Connect). The amplification conditions were as follows: Stage 1: Pre-denaturation 95°C, 30 s; Stage 2 (40 cycles): Denaturation 95°C, 15 s; Annealing 60°C, 30 s; Stage 3: 65°C, 5 min. The relative level of miRNA expression was analyzed by the 2^−ΔΔCT^ method. Each assay was performed in triplicate. The primer used are as follows (Table 1).

**TABLE 1:** The primers used for PCR.

Gene	Primer sequences
mmu-miR-132-RT	5′-CTC​AAC​TGG​TGT​CGT​GGA​GTC​GGC​AAT​TCA​GTT​GAG​CGA​CCA​TG-3′
mmu-miR-132	Forward:5′-ACACTCCAGCTGGGTAACAGTCTACAGCCA-3′
	Reverse:5′-TGGTGTCGTGGAGTCG-3′
U6	Forward:5′-CTCGCTTCGGCAGCACA-3′
	Reverse:5′-AACGCTTCACGAATTTGCGT-3′

### 2.4 *In Vitro* cell responses of HUVECs to miR132-Exo

The cell proliferation induced by miR132-exo was assessed by EdU analysis using an EDU-594 kit. In brief, HUVECs were seeded in a 6-well plate at a concentration of 10^5^/mL. After cell adhesion, equal volumes of miR-132-exo or Exo were added at a concentration of 2 μg/μl. The culture medium was changed 24 h later and co-cultured with EdU working solution (1:1,000) at 37°C for 3 h. The Click reaction solution was then added to each well and incubated at room temperature in the dark for 30 min. After washing, all cores were Hoechst stained. Images were captured by fluorescence microscopy and quantified by ImageJ. The specific methods are as follows: Firstly, the nuclei were selected and the number of nuclei in each field (Hoechst fluorescence image) was calculated by ImageJ software, which represented the number of all cells in the field and was named At. The number of green fluorescent cells in each field (EdU fluorescence image) was also calculated by mage J software and named A.The ratio of EdU positive cells was calculated as follows: EdU positive cell ratio (%) = A/At × 100%, and the cell proliferation ability was compared.

To assay the *in vitro* wound healing ability of miR-132-exo, cell scratch assay was performed. HUVECs were planted in 24-well plates (5 × 10^5^ cells/well) for 24 h. Next, cell surface was scratched in a straight line with a 200 μL pipette tip to generate wounds in each well, followed by PBS washing to remove cell debris. The miR-132-exo, exo or PBS were added to the wells for 24 h co-incubation at a concentration of 2 μg/μl. Subsequently, the scratched region was then photographed using a Leica microscope at the given time intervals (0, 24 , 48 h). The scratch closure was analyzed by the ImageJ soft-ware. The percentage of wound closure was calculated as follows: migration area (%)=(B0–Bn)/B0 × 100%, where B0 represents the initial wound area at 0 h and Bn represents the wound area at the time of measurement (24 and 48 h).

The cell migration was also assessed by a transwell migration assay. The HUVECs were cultured in the upper chamber, and miR-132-exo, Exo (2 μg/μl), or PBS were added to the lower chamber. After incubation at 37°C for 12 h, the membrane with migrated cells were fixed in 4% paraformaldehyde for 15 min and stained by 1% crystal violet in the dark for 30 min. The non-migrated side was carefully removed with a wet cotton swab, and the stained migrated cells were counted under a microscope.

### 2.5 Animal experiments

Animal Care and Use Committee at Wenzhou Medical University approved all animal procedures. Male C57BL/6 mice aged 6 weeks were obtained from Beijing Weitonglihua Laboratory Animal Technology Co., LTD. Prior to any experimental treatments, all C57BL/6 mice were kept under regular conditions and given food and water for 1 week. Mice were given a single intraperitoneal injection of 110 mg/kg streptozotocin (STZ, Sigma Aldridge) in citrate buffer to create a diabetic model (pH 4.5). At the end of the first 2 weeks, the weight and blood glucose levels of the mice were checked. Mice were diagnosed with diabetes when their glucose levels continuously surpassed 16.8 mmol/L, in conjunction with weight loss and polyuria symptoms. In order to determine the effective injection concentration, we did a preliminary experiment using 6 mice in each animal model and in the preliminary experimental design, we determined the injection concentration (2 μg/ml) for the experiment according to references (Shi et al., 2020) and it showed effective healing in both animal models. All diabetic mice were operated to create full-thickness skin wounds or free skin flaps on their back. In brief, mice were anesthetized with 1% sodium pentobarbital (50 mg/kg), and their shaved backs were sterilized with iodophor solution. Two round full-thickness wounds (1 cm in diameter) were made with a skin punch on the back of mice. Total thirty mice were used and randomly divided into three groups: control (normal saline), Exo, and miR-132-exo group. On day 0, 3, and 7, four injection points around the wound in each group were injected subcutaneously. The miR-132-exo group was injected with exosomes transfected with miR-132 (2 μg/μl, 25 μl for each injection point), and the Exo group was injected with pure exosomes (2 μg/μl, 25 μl for each injection point). The mice in control group were injected with equal volume of saline (25 μl for each injection point). At day 0, 3, 7, and 14, wound pictures were taken and wound area was further calculated by ImageJ software. The wound area percentage was determined as follows: (At/A0) × 100%, whereas A0 and At meant the wound area on day 0 and day 3, 7, and 14, respectively. A laser Doppler flowmeter was also used to scan the blood flow from wounds during healing and the results were quantified by MoorLDI Review software (ver.6.1; Moor Instruments). The collected tissue sections at certain time points were then used for H&E staining, immunohistochemistry (IHC) or immunofluorescence analysis.

In addition, another 30 mice were used to created random skin flaps (1.5 × 4.5 cm) on the back of mice and the bilateral subcutaneous trophoblast arteries providing blood supply of the flap pedicles were excised. Mice were also randomly divided into three groups: control, Exo, and miR-132-exo group. At day 0 and 3, the miR-132-exo, Exo or saline were injected subcutaneously at 8 injection points on each flap with the same dose and concentration as above. Then, the effect of relevant treatments on angiogenesis and flap regeneration was evaluated, and the flap tissue was further examined by histology experiments. High-quality random flap images were taken on the 0, 3, and 7 days respectively. According to the above images, ImageJ software was used to calculate the area of survival and area of necrosis. The necrotic area of the flap was defined by color change (appearance of a dull color), eschar, and non-epithelialized area. Survivable area percentage: survivable area × 100%/total area. A laser doppler flowmeter was used to assess the blood flow of the flap during the healing process, and the tissue of flap area II (at the junction of necrosis) was extracted for further study. The water content of specific flap tissue was manipulated as follows: on day 7, three flap tissues were taken from each group and weighed to calculate the “wet weight.” Next, these flaps were placed in an autoclave (50°C) and weighed daily until the weight remained stable for more than 2.days, at which point the recorded weight was considered “dry weight.” Tissue edema was calculated by the formula: ([wet weight—dry weight]/wet weight) × 100%.

### 2.6 Histological analysis, immunohistochemical staining and immunofluorescence staining

The collected wound or flap samples were fixed with 4% PFA for 3 days and then dehydrated in graded ethanol solutions (75%, 85%, 95%, 95%, 100%). The samples were then embedded in paraffin and cross-sectioned into 4-μm thickness slices. Standard H&E staining and Masson’s trichrome were performed. After sealing, the samples were observed and analyzed by a Nikon microscopy. The collagen-positive area and total tissue area of tissue sections were calculated by the statistical method of ImageJ software. Collagen deposition fraction = (collagen positive blue area/total tissue area) × 100%.

As for immunohistochemical staining, the rehydrated samples were immersed in 3% H_2_O_2_ for 10 min to block the endogenous peroxidase activity and then heated in 10.2 mM sodium citrate buffer (20 min, 95^°^C) for antigen retrieval. After blocking with 10% (w/v) bovine serum albumin (BSA)/PBS for 1 h, samples were then incubated with collagen I (1:200, Proteintech), collagen III (1:200, Proteintech), TNF-α (1: 200, Proteintech) or IL-6 (1: 200, Proteintech) primary antibodies at 4°C overnight. Later, the slides were incubated with secondary antibodies for 1 h at room temperature. The binding sites were then visualized with a DAB detection kit. All the samples were viewed under a microscope. The number of new vessels was determined by observing three random portions from each group. In addition, the IPP software measured the integrated optical density (IOD) of each field’s positive protein.

For immunofluorescence staining, the deparaffinized samples were rehydrated, heated and permeabilized with 0.1% (v/v) PBS/Triton X-100 for 20 min and blocked in BSA/PBS for 1 h. The samples were then incubated with α-SMA (1:100, Proteintech), Fibr (1:100, Proteintech), a mixture of α-SMA (1:100, Proteintech) and ZO-1 (1:100, Proteintech), a mixture of CD86 (1:100, Proteintech) and CD206 (1:100, Santa Cruz), a mixture of INOS (1:1,000, Abcam) and ARG-1 (1:100, Proteintech) or a mixture of α-SMA (1:100, Proteintech) and CD31 (1:100, Servicebio). After being rinsed with PBS, slices were incubated with Alexa Fluor 488- or Alexa Fluor 594-conjugated secondary antibodies (1:200, Abcam) for 30 min at room temperature. The nuclei were stained with mounting solution containing DAPI. The slides were examined using ImageJ software using a Nikon confocal laser microscope (A1 PLUS; Nikon, Tokyo, Japan).

### 2.7 Western blotting

Exosome protein extraction kit (Invitrogen) and BCA protein assay kit (Beyotime, Shanghai, China) were used to measure the concentration of exosomes and tissue protein. The protein samples (40 μg) were initially separated using SDS-PAGE before being transferred to a PVDF membrane (Bio-Rad, United States). After incubating with skim milk at a concentration of 5% for 2 hours to suppress the non-specific protein, the sample was then washed three times with TBST. The membranes were then incubated with primary antibodies against IKB Alpha (1:2000, Proteintech), phosph-IKB Alpha (1:2000, Affinity), NF-kb p65 (1:2000, Proteintech), phosph NF-kb p65 (1:2000, Affinity), TSG101 (1:1,000, Abcam), CD63 (1:1,000, Abcam)、CD81 (1:1,000, Abcam) overnight at 4°C, followed by incubation with the corresponding secondary antibody for 2 h. After rinsing, the binding site was treated with Electrochemiluminescence Plus reagent (Invitrogen), and image lab 3.0 software was used to quantitatively assess the band intensity (Bio-Rad), the data of target protein has been normalized to the GAPDH level through image lab 3.0 software.

### 2.8 Statistical analysis

All data are described as the mean ± standard deviation (SD). Comparisons among the groups were performed using one-way ANOVA with Tukey’s multiple comparison test. All analyses were performed with GraphPad Prism 9 software (GraphPad Software Inc., United States). In all tests, statistical significance was set at ∗*p*-value <0.05, ∗∗*p*-value <0.01 and ∗∗∗*p*-value <0.001.

## 3 Experimental results

### 3.1 Characterization of exosomes and miR-132-exo

**FIGURE 1:**
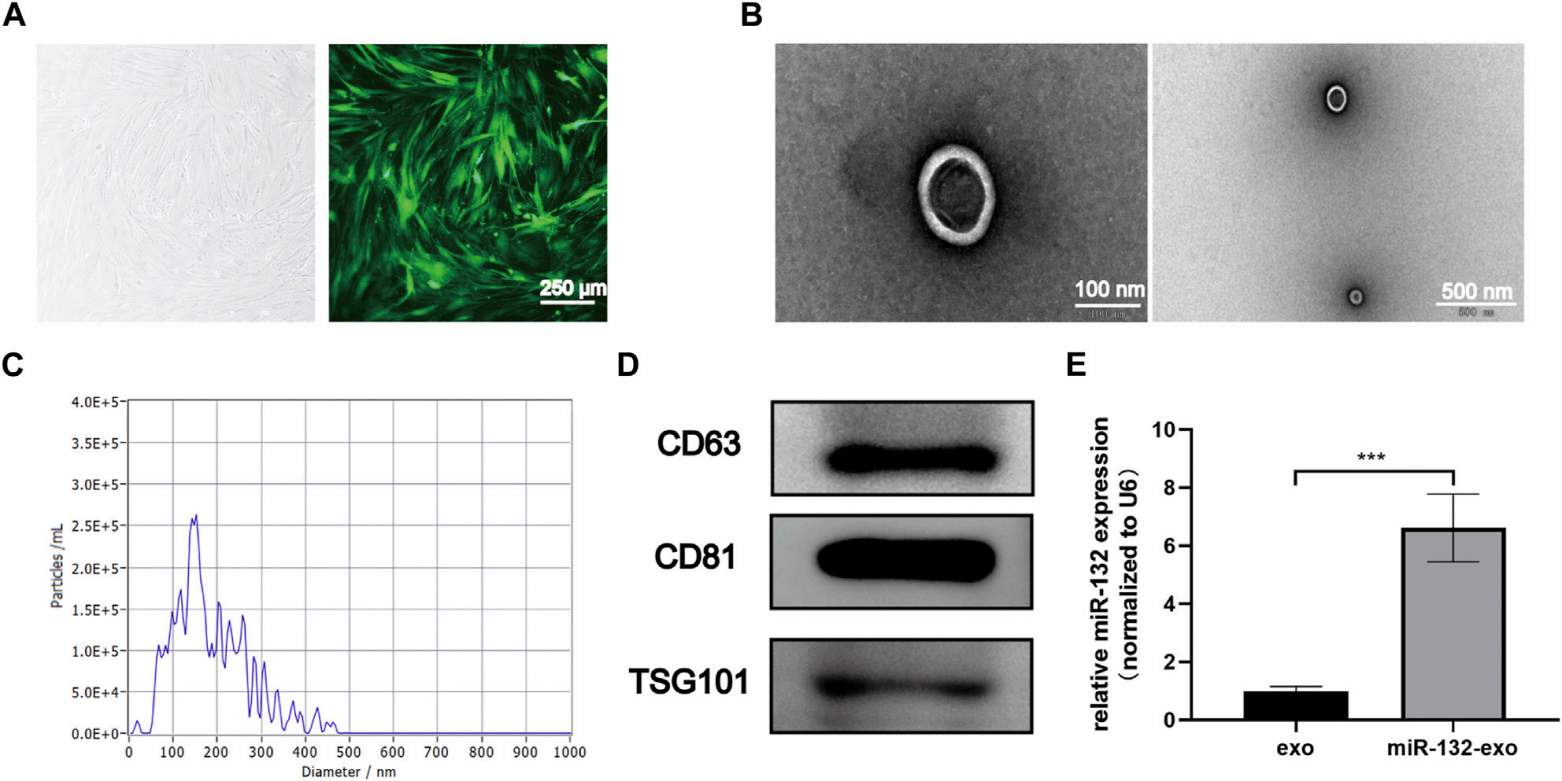
Characterization of ADSCs derived exosomes and the transduction efficiency. **(A)** The typical cobblestone-like morphology of ADSCs (left), and the morphology of ADSCs transfected with lentivirus labled with green fluorescent protein (GFP) (right); **(B)** The transmission electron microscopy (TEM) image of ADSCs-Exo (Scale bar = 100 nm, 500 nm); **(C)** Size distribution of exosomes by nanoparticle tracking analysis (NTA); **(D)** Western blot analysis of exosomes’ specific markers of CD63, CD81, and TSG101; **(E)** Loading efficiency of the Lentivirus in ADSCs derived exosomes calculated by qRT-PCR; miR-132 were used as a positive control, and expression was normalized to the housekeeping gene U6. *p < 0.05, **p < 0.01, ***p < 0.001, data are presented as the mean ± SD, *n* = 3.

The cultured adipose mesenchymal stem cells exhibited typical spindle shaped morphology (Figure 1A). The exosomes extracted through ultracentrifuge were characterized by TEM, NTA and Western blot analysis. TEM results showed that the naturally isolated exosomes exhibited spherical shape in morphology (Figure 1B). NTA data showed that the size of exosomes ranged from 80 to 150 nm (Figure 1C). In addition, Western blot results showed these exosomes exhibited high expression of the exosomal marker proteins, including CD63, CD81, and TSG101 (Figure 1D). These results indicated that exosomes derived from ADSCs were successfully extracted and obtained in this study.

The exosomes derived from miR-132 enriched ADSCs were also isolated. The qRt-PCR results showed that exosomes derived from ADSCs transfected with miR-132 expressed almost 5 times higher level of miR-132 than control, indicating that miR-132 has been successfully loaded into exosomes (Figure 1E).

### 3.2 Effect of miR-132-exo on proliferation and migration of HUVECs

**FIGURE 2:**
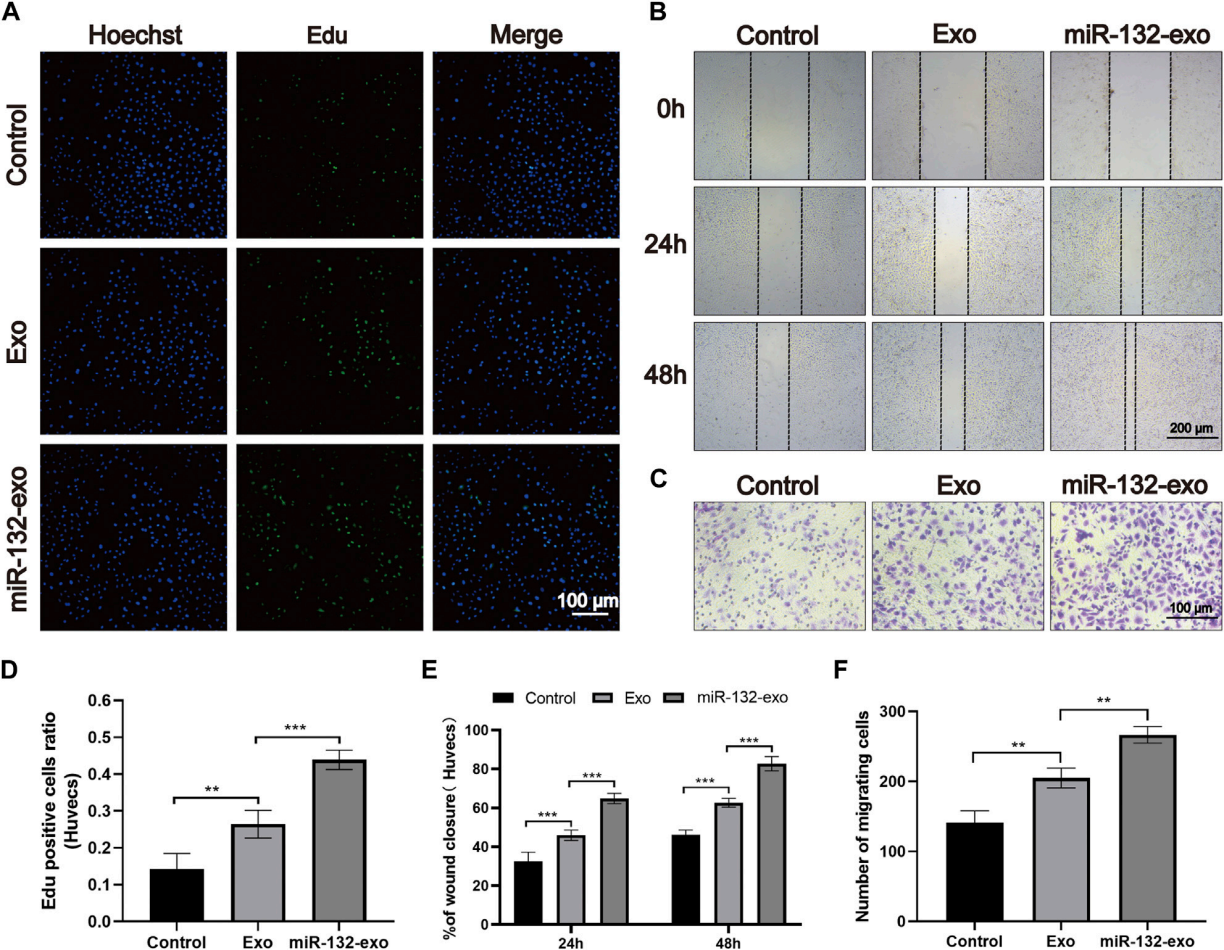
The effect of miR-132-exo on the proliferation and migration of HUVECs *in vitro*. **(A)** Representative fluorescence images of EdU staining of HUVECs treated with PBS, exosomes, and miR-132-exo for 24 h (scale bar = 100 μm); **(B)** Scratch wound assay of HUVECs following treatment with PBS, exosomes, and miR-132-exo at 0, 24 and 48 h (scale bar = 200 μm); **(C)** Representative transwell migration assay images of HUVECs treated with PBS, exosomes, and miR-132-exo for 12 h (scale bar = 100 μm); **(D)** Quantitative analysis of the EdU staining for proliferation rates in each group; **(E)** Quantitative data of the percentage of scratch wound closure in each group; **(F)** Quantitative analysis of the migrated cells HUVECs in each group. *p < 0.05, **p < 0.01, ***p < 0.001, data are presented as the mean ± SD, *n* = 6.

The effect of miR-132-exo on proliferation of endothelial cells was evaluated by EDU assay. As shown in Figures 2A,D, the average edu-positive cell rate of the control group was 14.26%, while that of Exo group and miR-132-exo group was 26.41% and 43.90%, respectively, it can be clear to see that both miR-132-exo and Exo promoted the proliferation of endothelial cells to a certain extent when compared with control, whereas the miR-132-exo exhibited the best effect on promoting proliferation of HUVECs.

The *in vitro* wound healing ability of miR-132-exo on HUVECs was evaluated by *in vitro* scratch test. All endothelial cells were incubated in a medium containing 1% FBS to eliminate the promoting effect of serum on cell proliferation. As shown in Figures 2B,E, after 24 h of cell scratch, the wound closure rate of control group was 33.45%. Compared with control, the Exo and miR-132-exo group showed statistically higher wound closure rates, which were 45.54% and 63.36%, respectively. After 48 h, this trend was more prominent. The wound closure rate of the miR-132-exo group raised up to 83.28% with shortest distance of scratch gap, while control group only achieved 46.22%, indicating miR-132-exo can enhance the cell migration ability of HUVECs. We used a transwell migration assay with crystal violet staining to evaluate cell movement *via* a transwell membrane, confirming that miR-132-exo can stimulate cell migration. The results (Figures 2C,F) also showed that the miR-132-exo group had the highest number of stained HUVECs that migrated, indicating that miR-132 can significantly promote cell migration ability of HUVECs. The above results indicated that miR-132-exo promoted the proliferation and migration of HUVECs *in vitro*, which should be beneficial for wound healing.

### 3.3 Effect of miR-132-exo on the survival of diabetic random skin flaps

**FIGURE 3:**
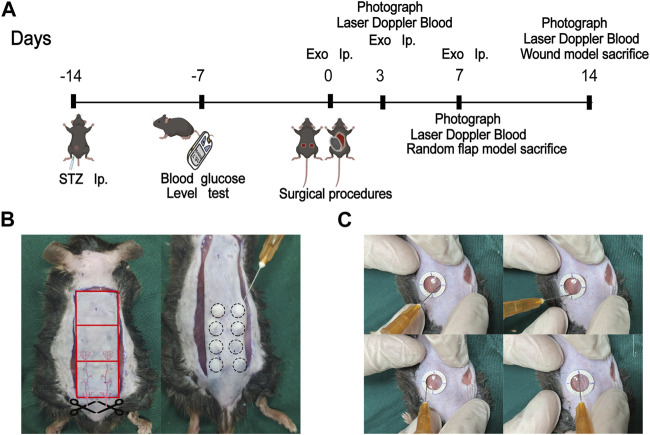
Schematic framework and animal surgery representative images. **(A)** The schematic framework of the animal experimental design; **(B)** The gross image of the construction of the random flap model (left) and the treatment of miR-132-exo on mice diabetic flap model (right); **(C)** The treatment of miR-132-exo on mice diabetic wound model.

**FIGURE 4:**
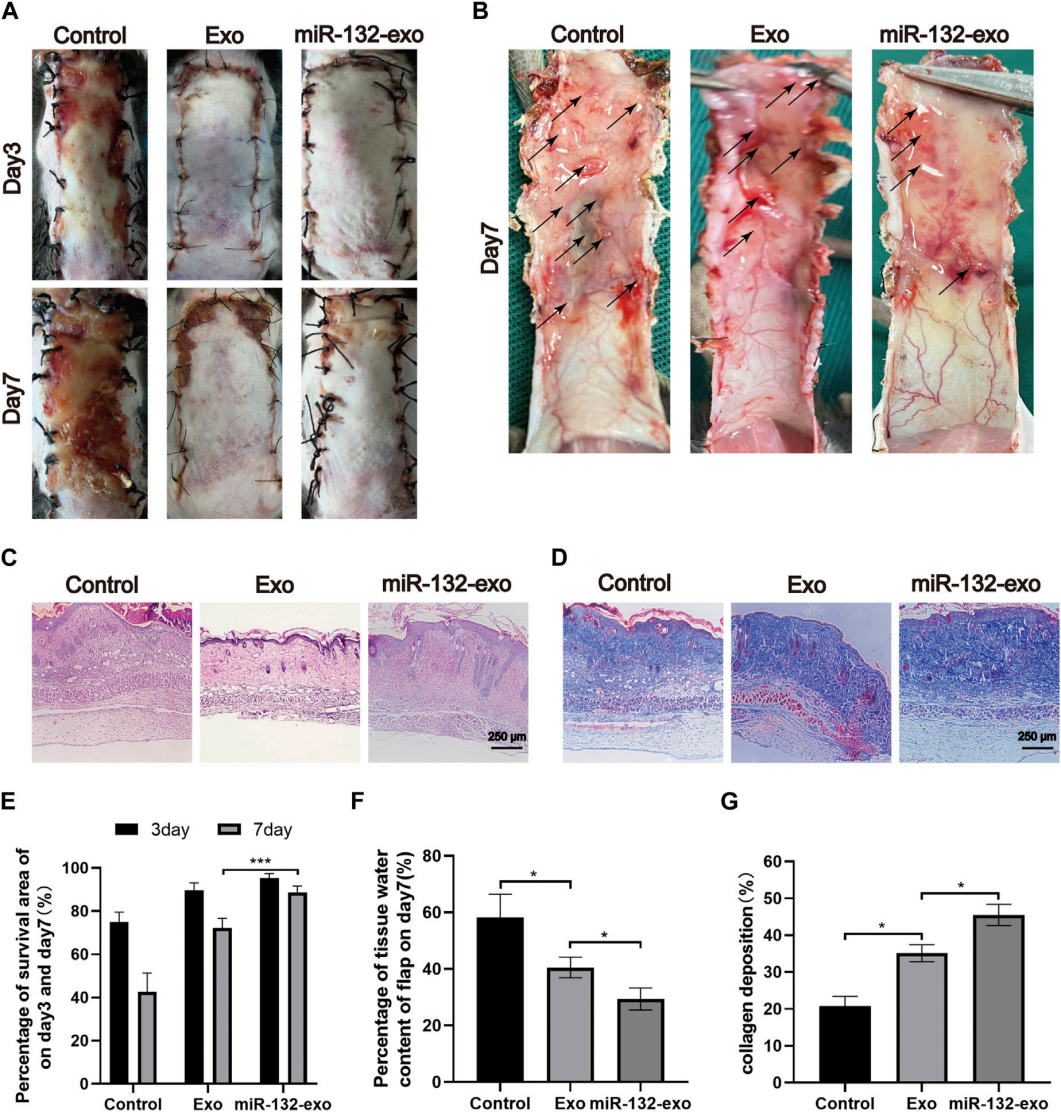
The miR-132-exo ameliorated the survival of diabetic random skin flaps *in vivo*. **(A)** Gross observation images of diabetic random skin flaps on the third and seventh day after treatment with normal saline, exosomes, and miR-132-exo; **(B)** Images reflected the tissue edema on postsurgery day 7, the edematous flap area is marked with a black arrow; **(C)** H&E staining images of the three groups on day 7; **(D)** Masson staining images of the three groups on day 7, scale bar = 250 μm; **(E)** Quantitative analysis of the percentage of survival area in three groups; **(F)** The percentage of tissue water content of the skin flaps in each group; **(G)** Quantitative analysis of collagen deposition in each group. *p < 0.05, **p < 0.01, ***p < 0.001, data are presented as the mean ± SD, n > 3.

STZ was injected intraperitoneally into mice to produce a model of diabetes. And a standard random skin flap model was established by surgery (Figure 3). The morphological changes of flap necrosis after 3 and 7days treatment (miR-132-exo, Exo or saline) were visually recorded by gross observation images. During the modeling process, the vascular pedicle was cut off, so the distal end part of the random flap was susceptible to ischemic necrosis. At day 3, we found small areas of necrosis began to appear in flaps, especially in control group, while the necrosis in miR-132-exo and Exo group was not noticeable. At day 7, different degrees of distal flap necrosis occurred in each group, whilst control group had the largest necrotic area. In contrast, the Exo and miR-132-exo treatment showed a much smaller necrotic area of the flaps, and the treatment of miR-132-exo was better than that of Exo (Figures 4A,E). These results indicated that miR-132-exo treatment can largely enhance the survival of diabetic skin flaps.

The edema of the flap tissue was measured to evaluate the survival rate of the flaps. Compared with control, the miR-132-exo group showed lower edema and subcutaneous venous congestion (Figure 4B). The Exo group also had a positive effect on decreasing the edema of the tissue compared with Control. The data of tissue water content at day 7 also confirmed that miR-132-exo treatment showed a statistical better effect on decreasing the edema extent and promote the survival of the flaps than Exo treatment (Figure 4F).

Hematoxylin-eosin (H&E) and Masson staining were also performed on the sections at the junction of necrosis and survival tissue at day 7. As shown in Figure 4C, compared with the control group, the Exo and the miR-132-exo group had prominent microvessels with complete skin structure and much fewer inflammatory cells, while the control group had a small number of fragile microvessels appeared at the bottom of the flap with the inferior blood supply and large numbers of inflammatory cells. Masson staining (Figures 4D,G) showed that the collagen fibers in the miR-132-exo group were densely arranged with order, while the collagen fibers in control were irregular and loose. All the above results confirmed that miR-132-exo facilitated the survival of diabetic random skin flaps *in vivo*.

### 3.4 Effect of miR-132-exo on promoting angiogenesis and vascular network formation in the random skin flaps

**FIGURE 5:**
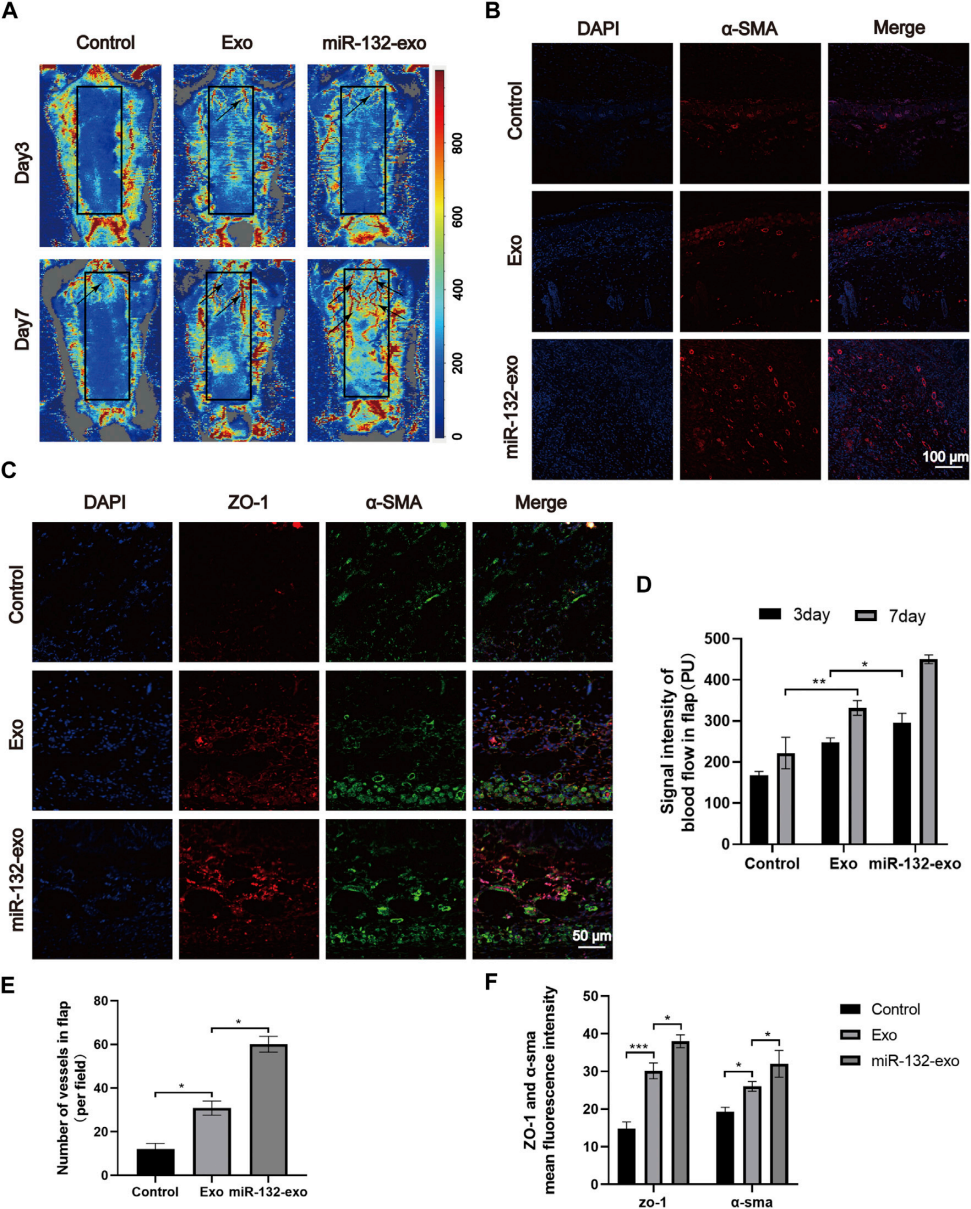
The miR-132-exo promoted angiogenesis and vascular network formation in diabetic random skin flaps. **(A)** Representative laser Doppler images on postsurgery day 3 and day 7; the arrows indicate the new blood vessels; **(B)** Representative immunofluorescence staining images of α-SMA-positive microvessels in each group on day 7, scale bar = 100 μm; **(C)** Representative immunofluorescence staining images of ZO-1 (red) and α-SMA (green) co-staining in each group on day 7, scale bar = 50 μm; **(D)** Quantitative data of signal intensity of blood flow in random skin flaps in each group; **(E)** Quantification of newly formed vessels per field corresponding to immunostaining of α-SMA; **(F)** Quantification of mean fluorescence intensity per field corresponding to co-staining of ZO-1 and α-SMA. *p < 0.05, **p < 0.01, ***p < 0.001, data are presented as the mean ± SD, *n* = 3.

Angiogenesis is essential for the survival of skin flaps. Therefore, the vascular status of the skin flaps was then evaluated by the blood flow laser Doppler equipment at day 3 and 7. As shown in Figure 5A, small amount of microvessels can be seen at the edge of wounds in miR-132-exo and Exo group at day 3, while no obvious newly-formed microvessels can been found in control group. With the increase of time, the miR-132-exo treatment significantly promoted the growth of microvessels in the flap pedicle and wound edge and increased the microvascular proliferation of the whole flap when compared with control. The blood flow signal (Figure 5D) data also confirmed that the flow intensity of the miR-132-exo group was significantly the highest among the three groups, followed by Exo and control group. The results indicated that the blood flow of the skin flaps can be restored by the miR-132-exo.

Meanwhile, α-SMA immunofluorescence staining was also performed to evaluate the angiogenesis of tissue flaps. The results in Figures 5B,E showed that the number of α-SMA labeled microvessels in miR-132-exo group was significantly higher than that in Exo and control groups. The newly formed microvessels helped deliver oxygen and nutrition to the distal end of the flaps and further benefited their survival. Tight junction protein ZO-1 is crucial for maintaining the mechanical barrier and permeability of epithelium. It not only participates in the regulation of cell material transport and maintenance of epithelial polarity but also plays an important part in cell proliferation and differentiation, which is closely related to the formation of new microvessels and the link between blood vessels. The ZO-1 and α-SMA co-staining (Figures 5C,F) showed that both of them were highly expressed in miR-132-exo group, indicating that miR-132-exo could promote the generation of neovascularization in flaps and the reconstruction of microtubules between vessels, which was more conducive to the generation of microvascular network.

Based on the above results, it can be inferred that miR-132-exo can upregulate the expression of ZO-1 and α-SMA and promote the angiogenesis and vascular network formation of the tissue, which restored the blood re-perfusion and further increased the survival rate of the random skin flaps.

### 3.5 Effect of miR-132-exo on stimulating M2 polarization of macrophages in diabetic random skin flaps

**FIGURE 6:**
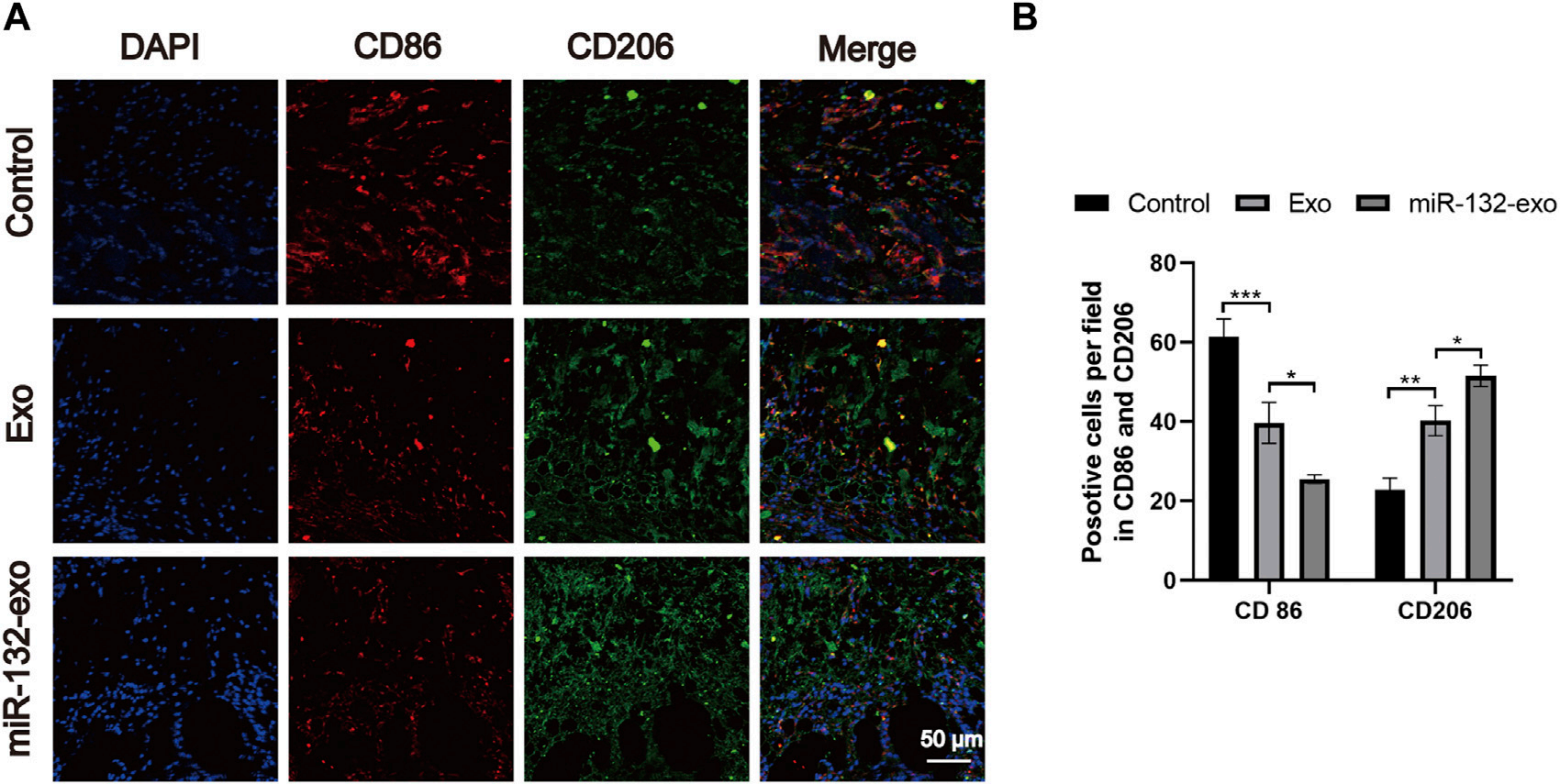
The miR-132-exo stimulated M2 polarization of macrophages in diabetic random skin flaps. **(A)** Representative co-staining images of CD86 and CD206 in each group, scale bar = 50 μm; **(B)** Quantification analysis of positive stained macrophages by CD86 and CD206. *p < 0.05, **p < 0.01, ***p < 0.001, data are presented as the mean ± SD, *n* = 3.

The flap regeneration is dependent on proper macrophage infiltration, and M2 macrophages are dominant and responsible for inflammation resolution and flap survival. We selected flap tissue sections of day 7, and co-stained CD86 (M1) and CD206 (M2). Figures 6A,B showed that more M2 macrophages and fewer M1 phenotype macrophages appeared in the miR-132-exo group. The results indicated that miR-132-exo can increase M2 macrophage infiltration during flap regeneration, further reduce inflammation and promoted the survival of the random skin flaps.

### 3.6 Effect of miR-132-exo on accelerating wound healing in diabetic mice

**FIGURE 7:**
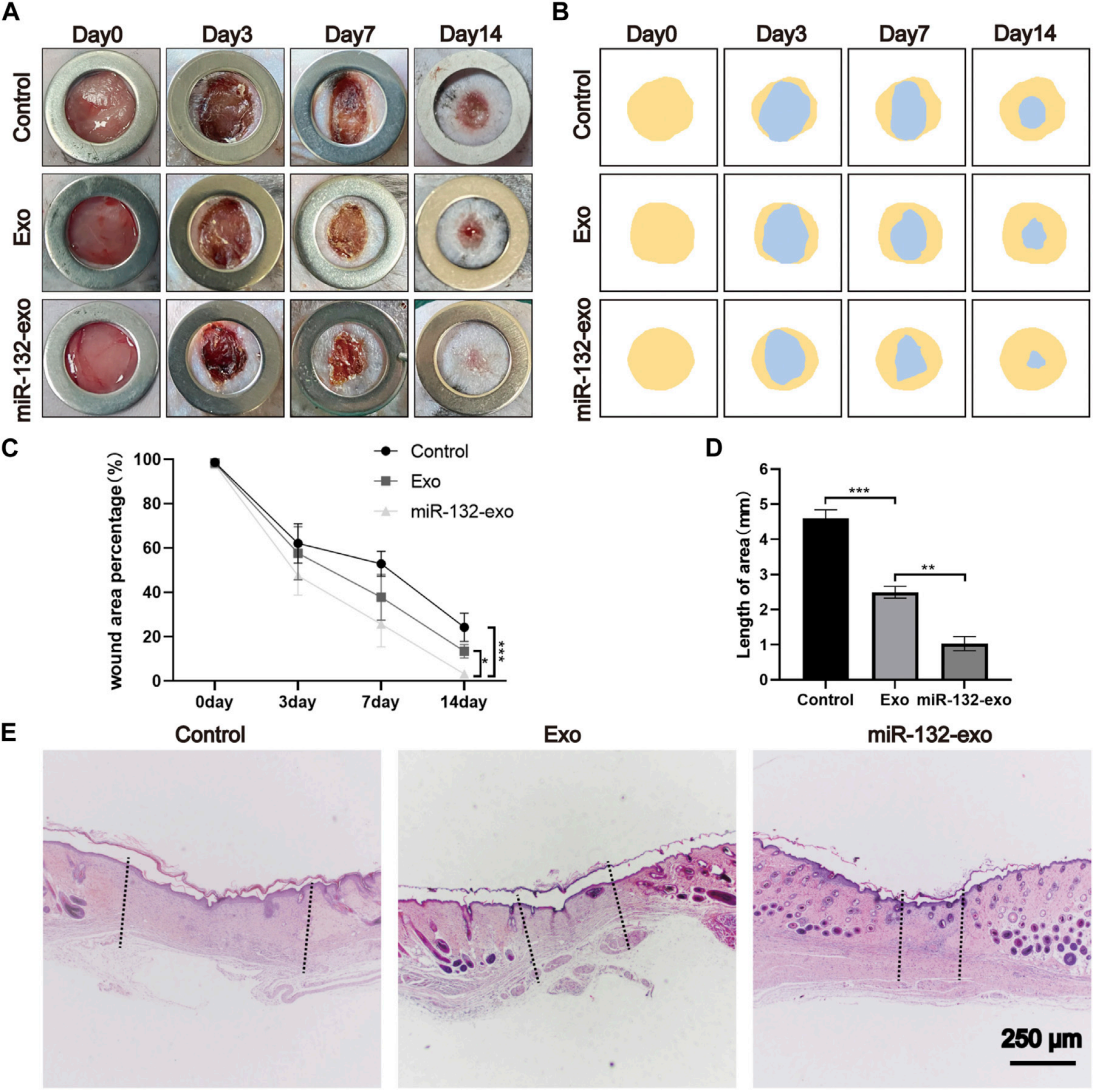
The miR-132-exo accelerated wound repair and regeneration in diabetic wounds. **(A)** Representative images of wound closure in a diabetic mouse model at the end of days 0, 3, 7, and 14 days following treatment with normal saline, exosomes, and miR-132-exo; **(B)** Schematic diagram of the wound area of each group; **(C)** Wound healing percentage of each groups at different time points; **(D)** Quantification of wound length assessed by H&E staining images in each group; **(E)** Representative images of H&E staining in each group on day 14, scale bar = 250 μm *p < 0.05, **p < 0.01, ***p < 0.001, data are presented as the mean ± SD, *n* = 6.

A mouse diabetic model was established by intraperitoneal injection of STZ, and a standard full-thickness skin defect model was created by surgery. Exo, miR-132-exo, or an equal volume of saline were injected subcutaneously around the wound and the morphological changes (Figure 7A) of wound healing at 0, 3, 7, and 14 days after operation were visually recorded. The blue area (Figure 7B) represented the wound size of each group on day 0, 3, 7, and 14. Although wound area decreased over time in all groups (Figure 7C), the difference in unhealed wound area between the three groups was not significant at day 3. However, at day 7 and 14, the area of unhealed wounds in Exo and miR-132-exo groups was significantly smaller than that in control group, which also indicated the strong potential of miR-132-exo in the treatment of diabetic wounds.

To further evaluate the healing pathology of the wounds, we selected a 14-day biopsy for H&E staining to assess epithelial formation, granulation tissue, and wound length. From Figures 7D,E it can be seen clearly that miR-132-exo group showed the smallest scar area with shortest wound lengths, followed by Exo group. Besides, miR-132-exo group also showed intact new epidermis with a clear and complex structure and more granulation tissue than control group.

### 3.7 Effect of miR-132-exo on promoting collagen deposition and ECM fibronectin hyperplasia in diabetic wounds

**FIGURE 8:**
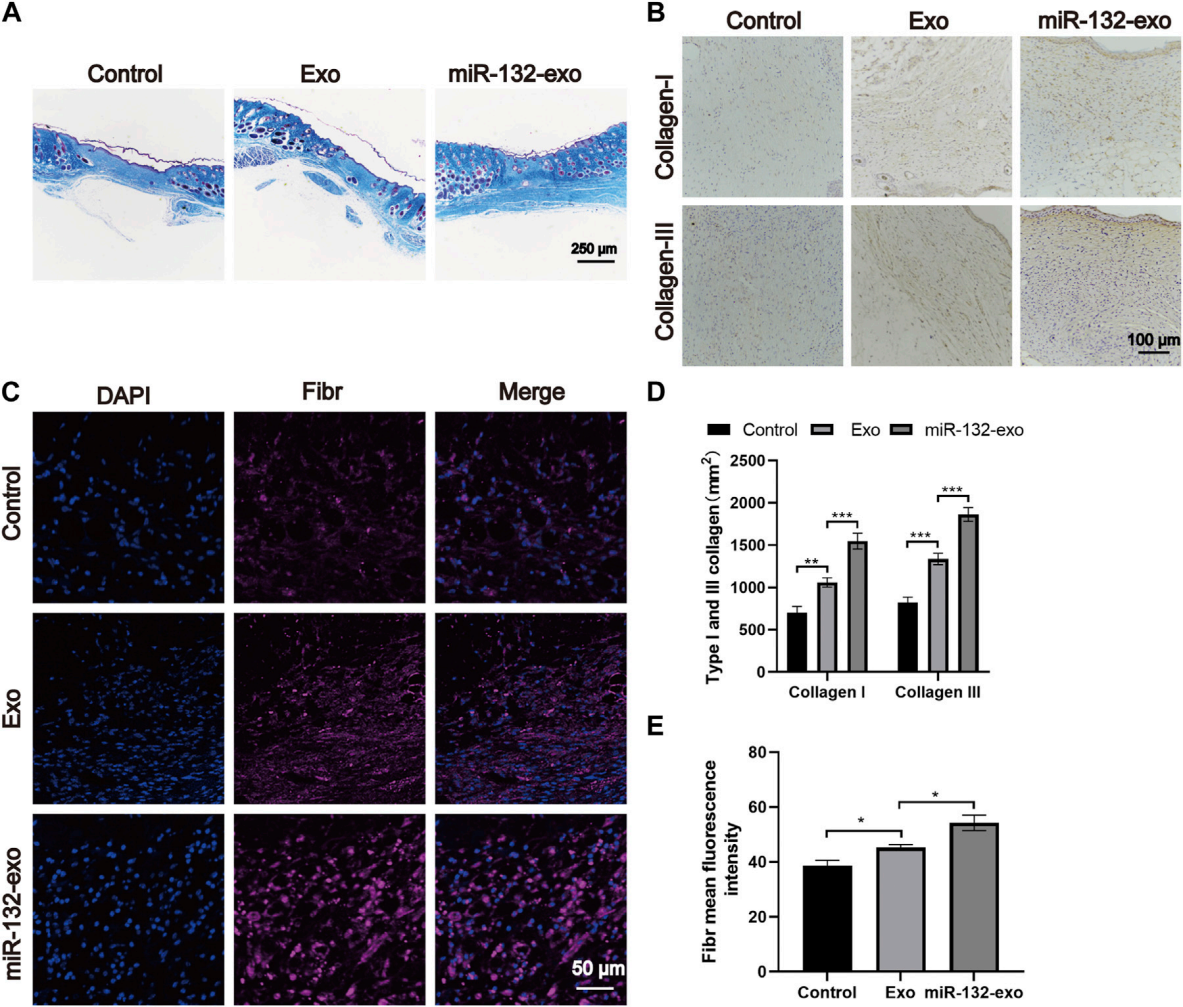
The miR-132-exo promoted collagen deposition and ECM fibronectin accretion in diabetic wounds. **(A)** Representative images of Masson staining in each group on day 14, scale bar = 250 μm; **(B)** Representative images of immunohistochemistry staining of collagen I and collagen III in each group on days 14, scale bar = 100 μm; **(C)** Representative images of Fibronectin immunostaining in each group on day 14, scale bar = 50 μm; **(D)** Quantitative data of relative density of collagen I and collagen III on days 14; **(E)** Quantification of mean fluorescence intensity per field of Fibronectin. *p < 0.05, **p < 0.01, ***p < 0.001, data are presented as the mean ± SD, *n* = 3.

The main components of the extracellular matrix are collagen, elastin, fibronectin, and other structural proteins. Hence, we select 14-day tissue sections for Masson trichrome staining to observe the collagen fibers in wound tissue. Figure 8A shows that all three groups had a wide distribution of blue color, but miR-132-exo group showed a darker blue color with more orderly arrangement than other groups, indicating that miR-132-Exo group had a faster collagen maturation with relatively well-arranged thick collagen fiber bundle. The COL I and COL III, which are the major collagen types in skin, are identified by immunostaining. As shown in Figures 8B,D, the positive staining of COL I and COL III in the miR-132-exo group were higher than in control, which are also consistent with the Masson staining results.

Fibronection is a multifunctional protein that is rich in the extracellular matrix (Brem and Tomic-Canic, 2007; Andreeva et al., 2016). In the process of dynamic wound healing and remodeling, Fibronection enhances the surrounding matrix formation with its fibrous structure. It can also be used as a biological glue to mediate the interaction between cells and other ECM components. The immunofluorescence staining of Fibronection in Figure 8C showed that the miR-132-exo group expressed more fibronectin, which could make various extracellular matrices and microvessels grow more closely and then benefit the diabetic wound healing.

### 3.8 Effect of miR-132-exo on promoting angiogenesis in diabetic wounds

**FIGURE 9:**
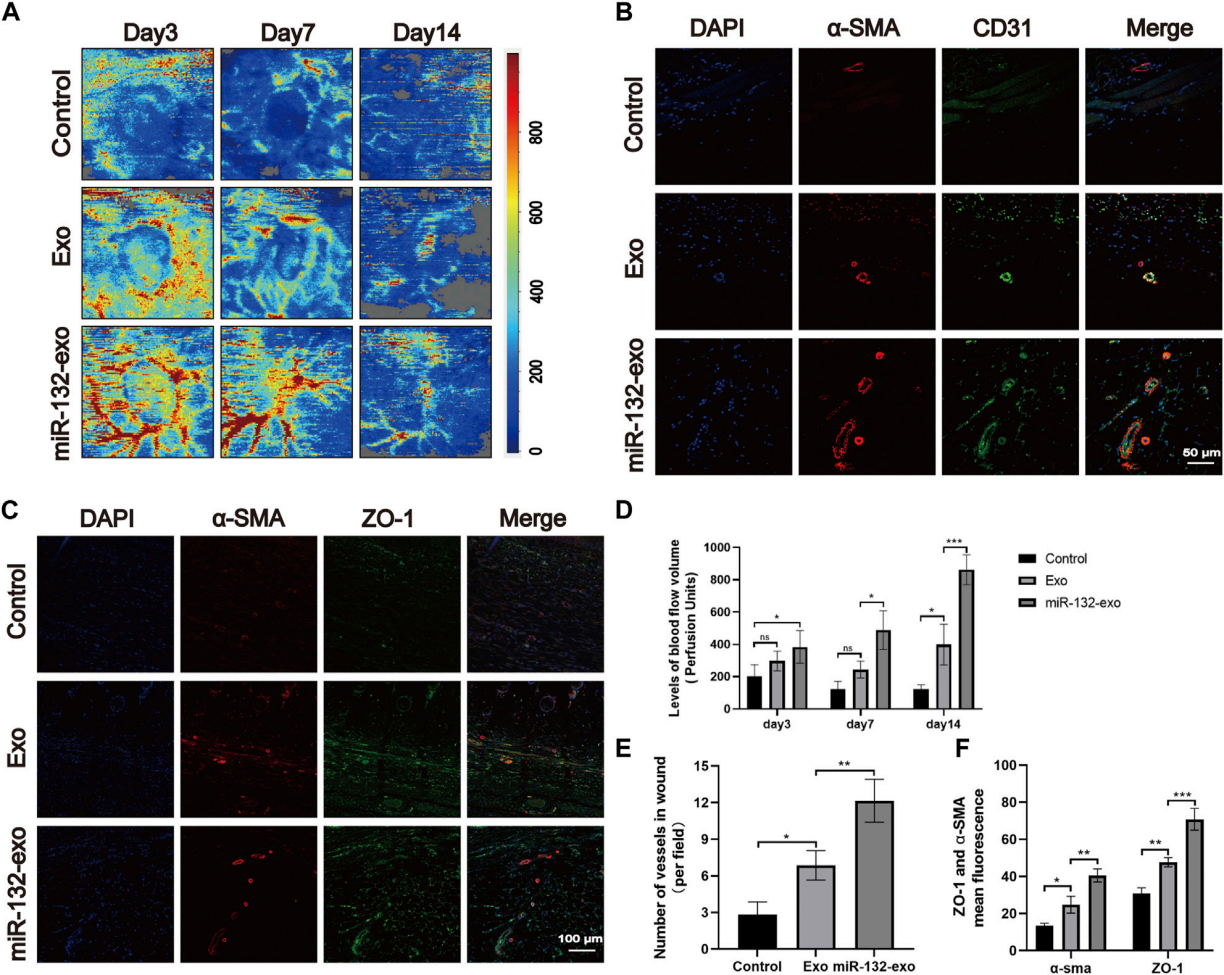
The miR-132-exo promoted angiogenesis and vascular network formation in diabetic wounds. **(A)** Representative laser Doppler images on postsurgery day 3, 7, and 14; **(B)** Representative co-staining images of α-SMA and CD31 positive microvessels in each group on day 14, scale bar = 50 μm; **(C)** Representative immunofluorescence staining images of ZO-1 (red) and α-SMA (green) co-staining in each group on day 14, scale bar = 100 μm; **(D)** Quantitative data of signal intensity of blood flow in diabetic wounds in each group; **(E)** Quantification of newly formed vessels per field corresponding to co-staining of α-SMA and CD31; **(F)** Quantification of mean fluorescence intensity per field corresponding to co-staining of ZO-1 and α-SMA. *p < 0.05, **p < 0.01, ***p < 0.001, data are presented as the mean ± SD, *n* = 3.

The angiogenesis is vital in wound healing due to the function of transporting nutrition and oxygen of blood vessels to the wound site. To further verify the role of miR-132-exo in promoting the process of wound neovascularization, Laser Doppler scanning imaging (Figure 9A) was used to evaluate the recovery of vascular bed in the whole flap area at Day 3, 7 and 14. The results (Figure 9D) showed that Exo group exhibited higher blood flow level than Control at day 7 and 14. However, even at the early time of healing (day 3), miR-132-exo group already presented the highest blood flow level among the three groups and lasted till day 14. Further, the immunofluorescence staining of the marker of vascular endothelial cells (CD31) and the marker of vascular smooth muscle cells α-smooth muscle actin (α-SMA) were performed. We found that CD31 and α-SMA (Figures 9B,E) were widely expressed in wound beds treated by miR-132-exo. Compared with control and Exo group, miR-132-exo group showed more new and mature blood vessels, which indicated that miR-132-exo stimulated the regeneration of blood vessels in diabetic wounds. Tight junction protein ZO-1 is one of the crucial proteins to maintain epithelial mechanical barrier and permeability. ZO-1 co-stained with α-SMA results (Figures 9C,F) indicated that miR-132-exo could promote tight junction between the new blood vessels in wounds, which are helpful for the generation of microvascular networks. These results further proved that miR-132-exo stimulated the angiogenesis process in diabetic wounds, which can contribute to the faster healing observed in the above gross observation results.

### 3.9 Effect of miR-132-exo on relieving inflammation and stimulating M2 polarization of macrophages in diabetic wounds

**FIGURE 10:**
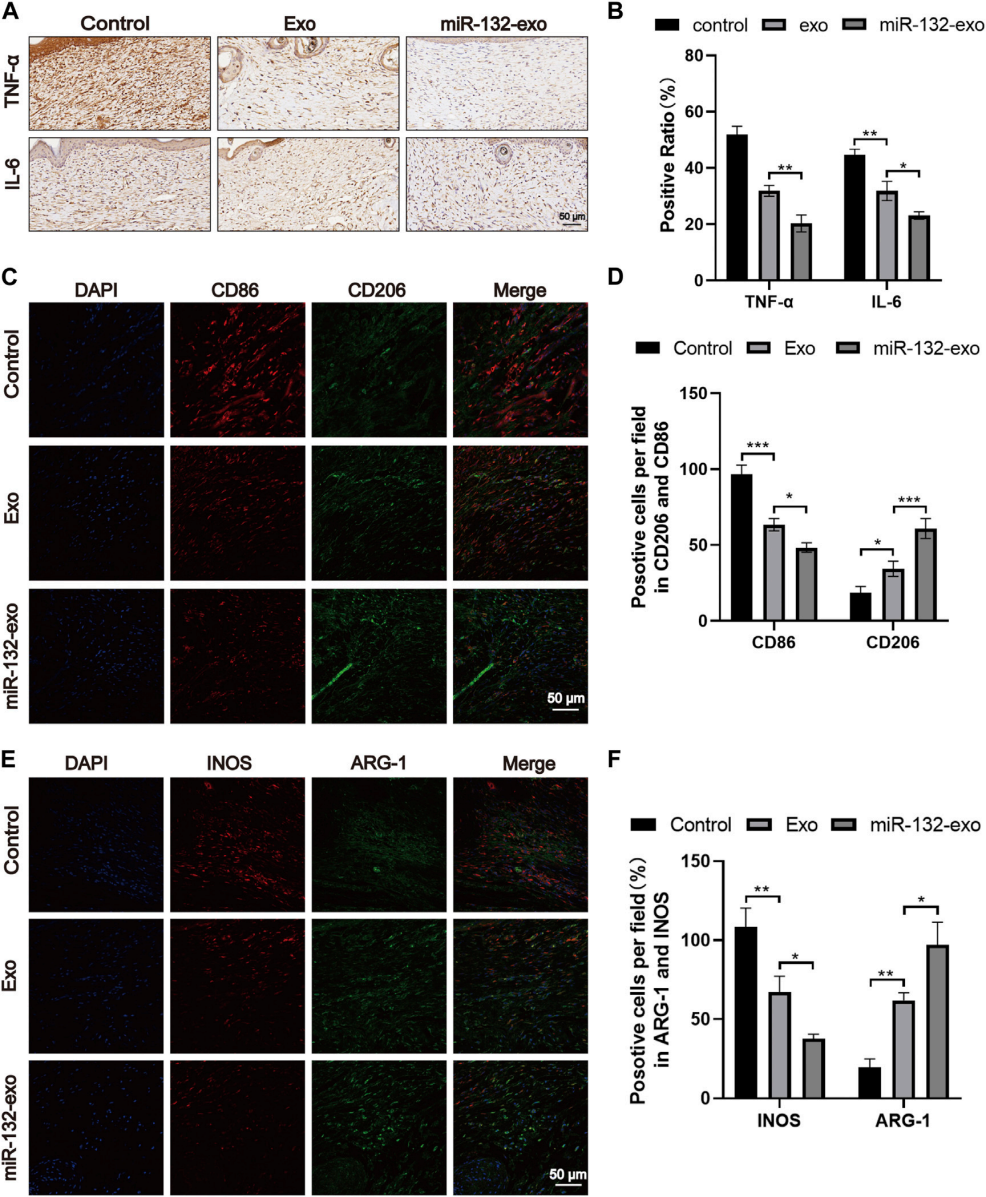
The miR-132-exo relieved inflammation and stimulated M2 polarization of macrophages in diabetic wounds. **(A, B)** Representative images **(A)** and positive staining ratio of immuostaining of TNF-α and IL-6 in each group on day 14, scale bar = 50 μm; **(C, D)** Representative images and quantification analysis of co-staining of CD86 and CD206 in each group on day 14, scale bar = 50 μm; **(E, F)** Representative images and quantification analysis of co-staining of INOS and ARG-1 in each group on day 14, scale bar = 50 μm *p < 0.05, **p < 0.01, ***p < 0.001, data are presented as the mean ± SD, *n* = 3.

Macrophages play an essential role in wound healing. M1 phenotype macrophages are usually involved in pro-inflammatory responses, while M2 macrophages are associated with anti-inflammatory responses. In the wound healing stage, the expression of IL-6 and TNF-α is the central link of inflammation and also reflects the severity of inflammation. The tissue immunostaining (Figures 10A,B) showed lower level of TNF-α and IL-6 when compared with Exo and Control group, indicating the inflammation in wound tissue treated by miR-132-exo was gradually reduced. In order to further study the relationship between inflammation and macrophage polarization, we selected wound tissue sections of day 14, and co-stained CD86 (M1) and CD206 (M2) using immunofluorescence staining. It was shown (Figures 10C,D) that more M2 macrophages and fewer M1 phenotype macrophages appeared in miR-132-exo group. The immunostaining of INOS (M1) and ARG (M2) (Figures 10E,F) also showed the similar trend. The results here indicated that miR-132-exo promoted the polarization of macrophages to M2 macrophages and had a good anti-inflammatory ability, which can reduce the inflammation in diabetic wounds and accelerate the healing process.

### 3.10 Effect of miR-132-exo on inhibiting the NF-KB pathway in diabetic wounds

**FIGURE 11:**
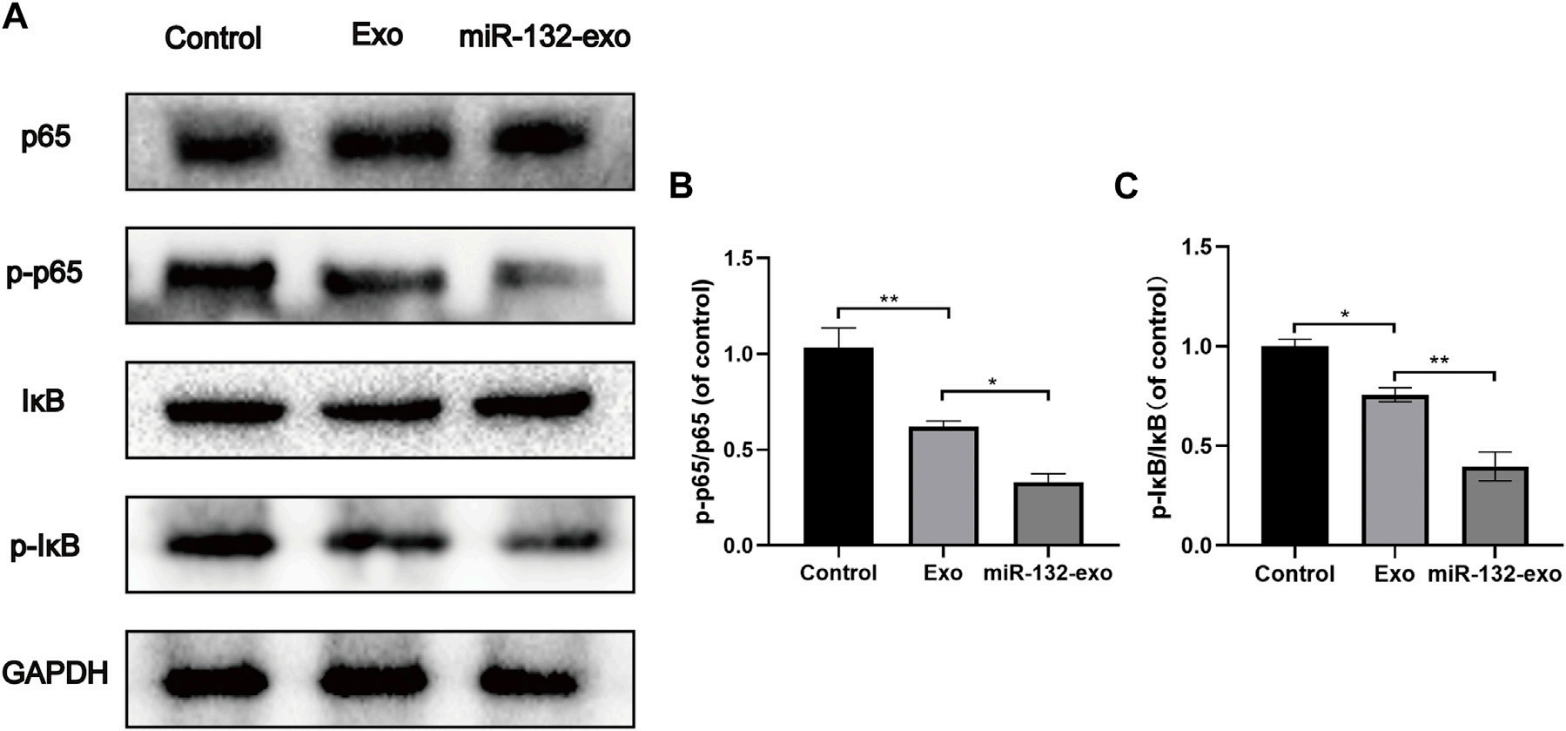
The miR-132-exo inhibited the NF-KB pathway in diabetic wounds. **(A–C)** Western blot bands and quantitative analysis of P65, PP65, lκB, P-lκB in each group. *p < 0.05, **p < 0.01, ***p < 0.001, data are presented as the mean ± SD, *n* = 3.

The mechanism by which miR-132-exo stimulating M2 macrophage polarization was also investigated. Western blotting results showed that the expression of p-p65 and p-IκB was lower in miR-132-exo group than that in control and Exo groups (Figures 11A–C), suggesting that the miR-132-exo treatment notably inhibited the phosphorylation of NF-κB p65 and IκBα in wound tissue of diabetic mice, thereby reducing the expression of inflammatory cytokines of TNF-α and IL-6. In conclusion, miR-132-exo may promote the polarization of macrophages and reduce the expression of inflammatory factors by inhibiting the NF-κB pathway, thereby reducing inflammation and promoting the transition from inflammation to proliferation in the process of wound healing.

## 4 Discussion

The diabetic wounds have become one of the main types of wounds in clinic. The impaired healing of diabetic wounds is mainly characterized by long-term severe inflammation, insufficient and low-activity growth factors, difficult to vascularization, excessive ROS, and easy infection (Goswami et al., 2022). Minor defects of diabetic skin can often be healed by drugs, growth factors, dressings, and other methods. In contrast, large-scale wounds need to be grafted with flaps, which usually face the risk of necrosis in diabetic wounds (Dolati et al., 2020). Therefore, new therapeutics that help the tissue reconstruction and the survival of skin flaps are highly needed for the treatment of diabetic wounds (Houlind, 2020). Previous studies have shown that exosomes derived from adipose stem cells has a good effect on promoting wound healing, and loading functional miRNA into exosomes can conquer some of the difficulties in diabetic wound healing (Wang et al., 2021). In this study, the engineered exosomes derived from miR-132-overexpressing ADSCs was constructed. Endothelial cells can have their proliferation and migration boosted by miR-132-exo. In addition, both the diabetic wound healing and the survival of random skin flaps were significantly improved by the miR-132-exo through alleviating inflammatory response and promoting angiogenesis, which were closely related to the higher level of M2-macrophages polarization mediated by inhibiting the NF-kB signaling pathway.

Reducing severe inflammation is critical to the healing outcomes of diabetic wounds (Aitcheson et al., 2021). In the process of wound healing, macrophages play an essential part in the inflammatory response (Liu et al., 2020a). According to their different phenotypes and functional plasticity, macrophages can be divided into M1-like phenotype macrophages and M2-like phenotype macrophages, whereas diabetes may cause the difficulty for the transition of M0/M1 macrophages polarizing into M2 macrophages and hinder the healing process (Kuo et al., 2021). In our study, to further determine the type of macrophages in skin flaps/wounds, the molecular marker of M1/M2 macrophages were labelled by CD86 or INOS (M1) and CD206 or Arg-1 (M2). We found that the miR-132-exo group showed more M2 macrophages than other two groups in damaged area, while control group showed much more M1 macrophages with few M2 macrophages, which indicated that miR-132-exo could promote the polarization of macrophages into the M2 phenotype during healing and tissue reconstruction. This may be due to the function of the enriched miR-132 in exosomes, which has been reported that it can induce the polarization of M2 macrophages and regulate the inflammatory response (Liu et al., 2020a). Then, we detected the expression of inflammation-related proteins in tissue, and the results of immunohistochemical staining of TNF-α and IL-6 showed that the miR-132-exo group has lower inflammation compared with control, which could be the result of the induced polarization of M2 macrophages by miR-132-exo (Kotwal and Chien, 2017). The H&E staining results also confirmed the reduced inflammatory response in miR-132-exo treated wounds. Hence, miR-132-exo can effectively reduce the excessive inflammation around the wounds and the transplanted skin flaps under diabetes condition, which presented great potential in promoting the survival of diabetic skin flaps and the regeneration of diabetic wounds.

Another important factor that controls the healing rate of diabetic wounds is the ability of angiogenesis and further vascularization in the regenerated tissue (Okonkwo and DiPietro, 2017). The newly-formed blood vessels can provide oxygen and nutrition for the metabolism of cells around diabetic wounds (Wang et al., 2018). Emerging studies have shown the angiogenic ability of exosomes derived from adipose stem cells (Chen et al., 2022). Besides, the advantage of exosomes’ natural availability and biocompatibility makes them an excellent tool for miR-132 transport for the precision therapy in diabetic wound repair and skin reconstruction (Yong et al., 2019; Joo et al., 2020; Zhou et al., 2020). It has also been found that miR-132 can regulate angiogenesis by inhibiting the NF-κB pathway and activate the VEGF pathway in ICD diseases (Che et al., 2018). In this study, miR-132-exo showed excellent pro-angiogenic ability both *in vivo* and *in vitro*. *In vitro* endothelial cell experiments proved that miR-132-exo could promote endothelial cell proliferation and migration, which is the beginning and fundamental of the angiogenesis process. The expression of CD31 and α-SMA showed higher numbers of newly-formed vessels in flaps and wound tissues treated by miR-132-exo, which was consistent with the laser Doppler results that clearly showed the higher blood flow in miR-132-exo group compared with exo and control during the regeneration of diabetic wounds and the skin flaps. Compared with control, the staining of tight junction protein ZO-1 also showed that miR-132-exo contributed to the interconnection and communication between microvessels and was more conducive to the communication of blood flow and the delivery of nutrients and oxygen (Kuo et al., 2021). This excellent angiogenic effect stimulated by miR-132-exo should be due to the synergetic effect of both miR-132 and the exosomes themselves, which also lays a foundation for the rapid formation of granulation tissue and epithelial re-formation in diabetic wounds (Shaabani et al., 2022). At the same time, this angiogenic ability of miR-132-exo also solved the problem of necrosis in the distal part of skin flaps cause by insufficient vascular circulation after transplantation, thus enhanced the survival rate of the skin flaps.

Due to the excellent performance of miR-132-exo in anti-inflammation and promoting angiogenesis, after the inflammatory stage, granulation tissue began to grow around diabetes wounds with an enormous number of cells, extracellular matrix, and new capillaries (Xu et al., 2015). The miR-132-exo group showed abundant granulation tissue and significantly shortened wound length, while the control group showed relatively less granulation tissue and ECM, resulting in a lack of matrix to facilitate effective communication between endogenous cells (Liu et al., 2022). With the increase of healing time, collagen fibers gradually replaced the granulation tissue. Neatly arranged collagen fibers were deposited in wounds treated with miR-132-exo with abundant type I and type III collagen in diabetic tissues, and this abundant extracellular matrix provides a good growth space for endogenous cells to grow (Liu et al., 2022). In the transplanted skin flaps, a more complete epithelial structure with abundant granulation tissue and arranged collagen fibers can be clearly found in miR-132-exo group, hence resulting in a faster and better healing results of the diabetic wounds/skin flaps.

In LPS-induced inflammation, the NF-κB signaling pathway is the most critical pathway for macrophage activation and polarization (Liu et al., 2020b). In this study, miR-132-exo significantly inhibited the phosphorylation of NF--κB p65 and IκB during diabetic wound healing, which indicated that miR-132-exo inhibited the NF-κB signaling pathway and further stimulated the M2 macrophages polarization to reduce the expression of inflammatory factors TNF-a and IL-6. This could reduce the inflammatory response in diabetic wounds and skin flaps and further accelerated wound repair and skin reconstruction.

In this study, the anti-inflammatory, pro-angiogenic, pro-healing, and the possible underlying mechanisms of miR-132-exo in promoting diabetic wound healing and the survival of random skin flaps have been identified. Due to the synergetic effect of miR-132 and exosomes, the miR-132-exo achieved good anti-inflammatory ability through inducing the M2-macrophages polarization, improved vascularization, and reached faster granulation tissue formation and collagen formation, together accelerated the diabetic wound healling and enhanced the survival of random skin flaps. This study mimicked two circumstances of diabetic wounds, the relatively small one and a large one that needs the skin graft, and miR-132-exo showed potent positive healing effect on both wounds. Taken together, miR-132-exo can be an excellent candidate in promoting the healing of diabetic wounds and skin flaps. In diabetic patients, it is worth noting that the chronic non-healing wounds involve more complex cross-talking among pathogenic agent, immune cells, tissue cells in the disease process compared with experimental models. Prior to preclinical testing, additional research is required to understand the exact mechanisms of miR-132-exo on diabetic wounds.

## 5 Conclusion

**FIGURE 12:**
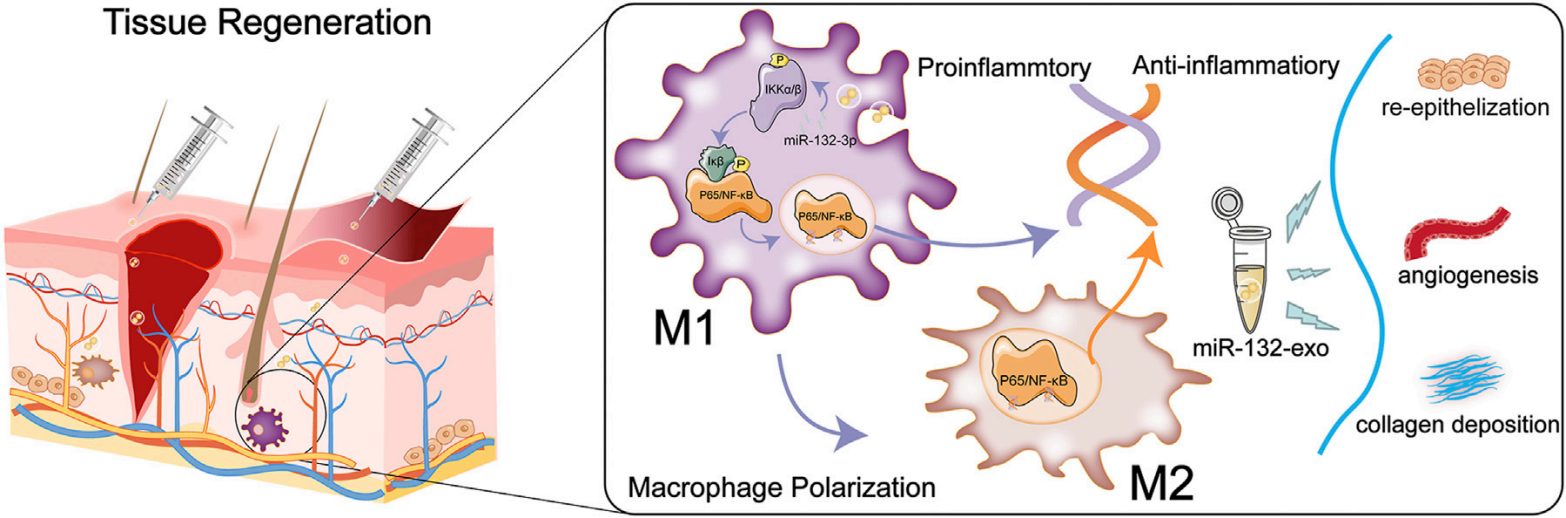
Schematic diagram of the function of miR-132-exo in wound repair and skin reconstruction and its possible mechanism.

In conclusion, we combined exosome therapy with precision delivery of miR-132 into adipose stem cell-derived exosomes for diabetic wound healing. The miR-132-exo can accelerate diabetic wound healing and enhance the survival of random skin flaps by inducing the M2-macrophages polarization, accelerating angiogenesis and vascularization, and increasing collagen remodeling. In addition, the M2 macrophages polarization induced by miR-132-exo may be mediated by inhibiting the NF-κB signaling pathway through suppressing the phosphorylation of p65 and IκB, which further reduced the inflammation and promoted the angiogenesis of random skin flaps and in wound tissue (Figure 12). These results indicated that engineered miR-132-exo can be an excellent candidate for precision treatment of diabetic wounds and other inflammatory-related disease.

## Data Availability

The raw data supporting the conclusion of this article will be made available by the authors, without undue reservation.
