# Supplementary material for: Engineered exosomes derived from miR-132-overexpresssing adipose stem cells promoted diabetic wound healing and skin reconstruction
Source: Front Bioeng Biotechnol. 2023 Mar 1;11:1129538. doi: 10.3389/fbioe.2023.1129538 (PMC10014603; doi:10.3389/fbioe.2023.1129538)

Figure1

Figure1A

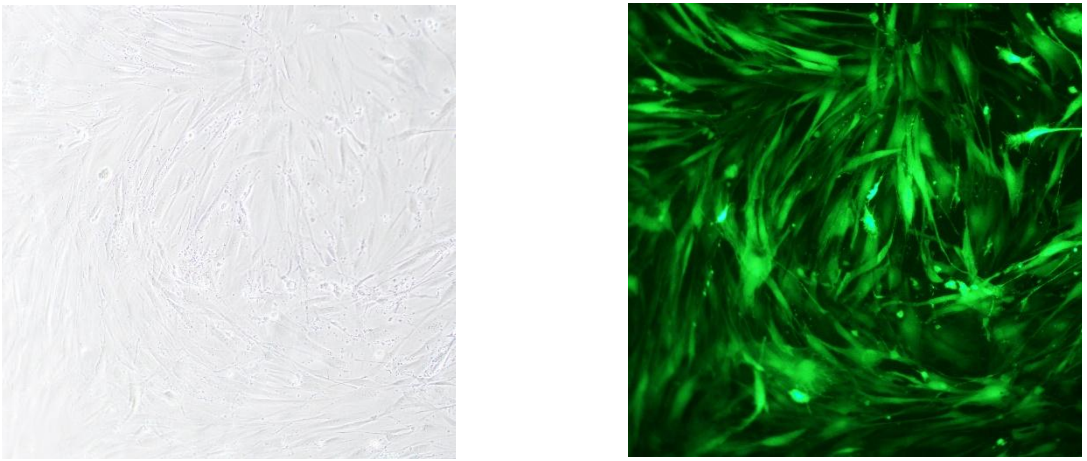

Figure1B

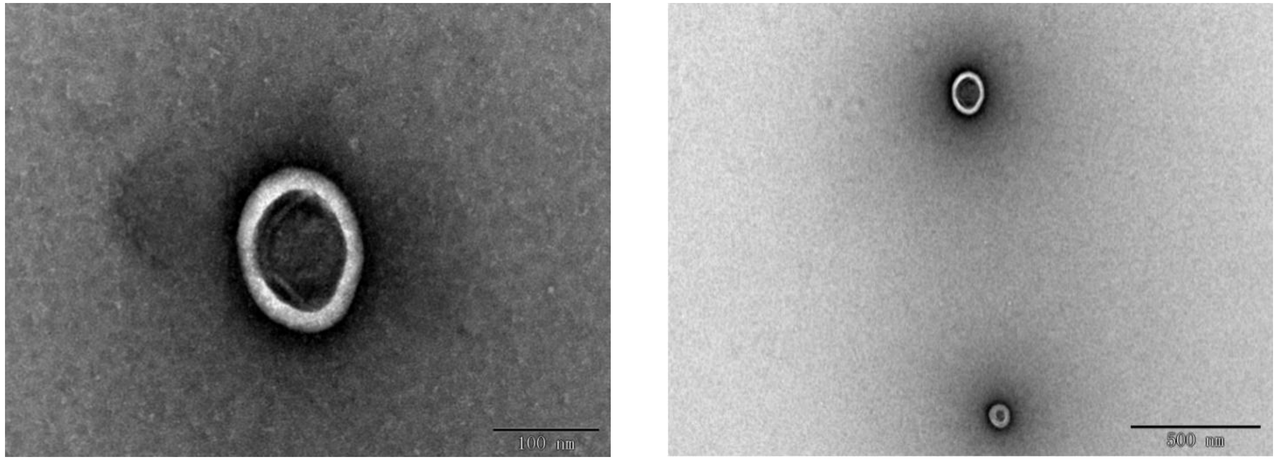

Figure1C

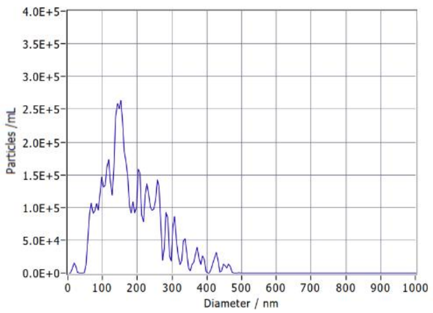

Figure1D

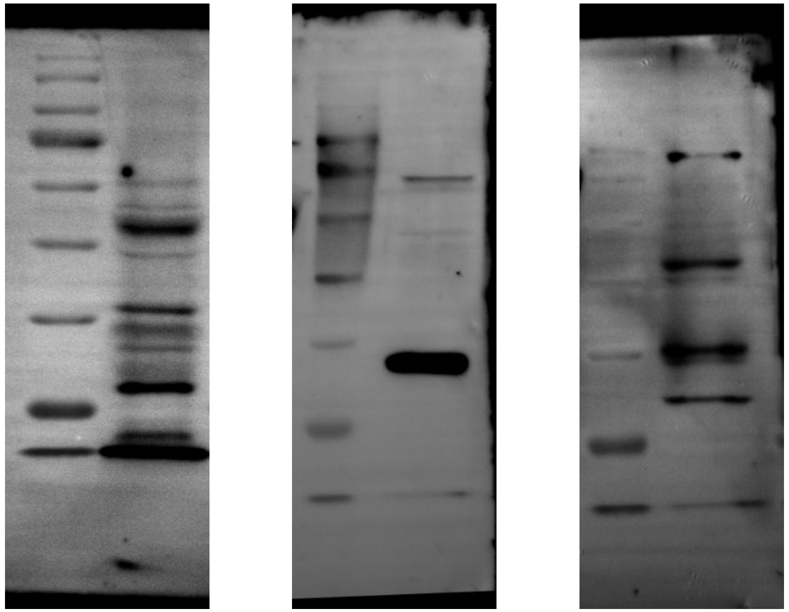

Figure2

Figure2A

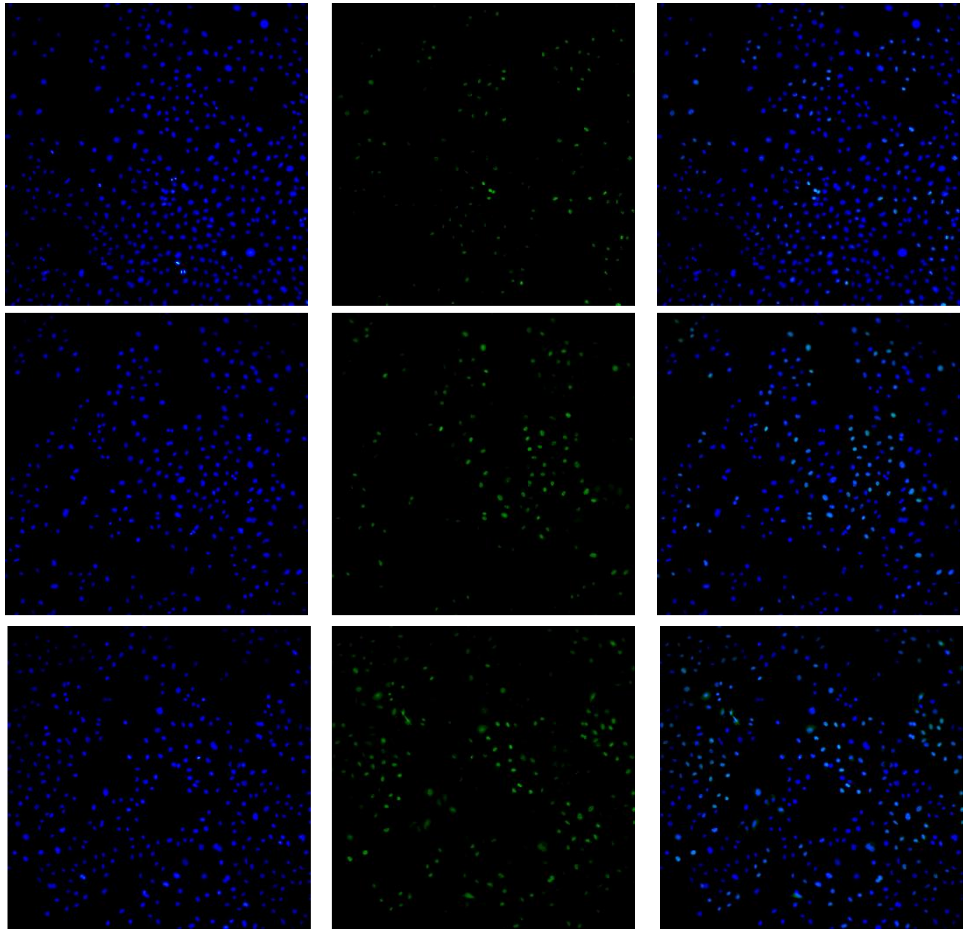

Figure2B

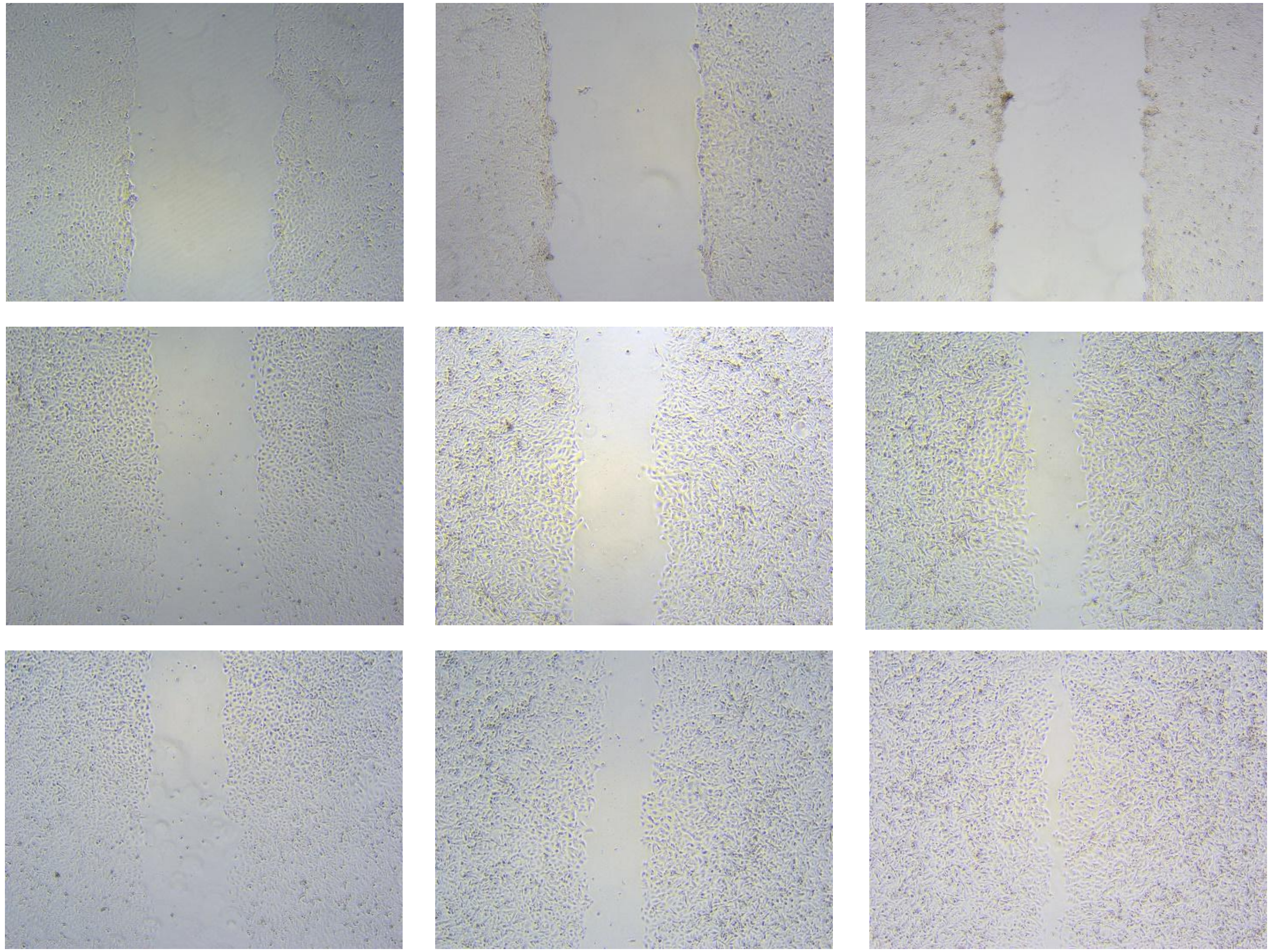

Figure2C

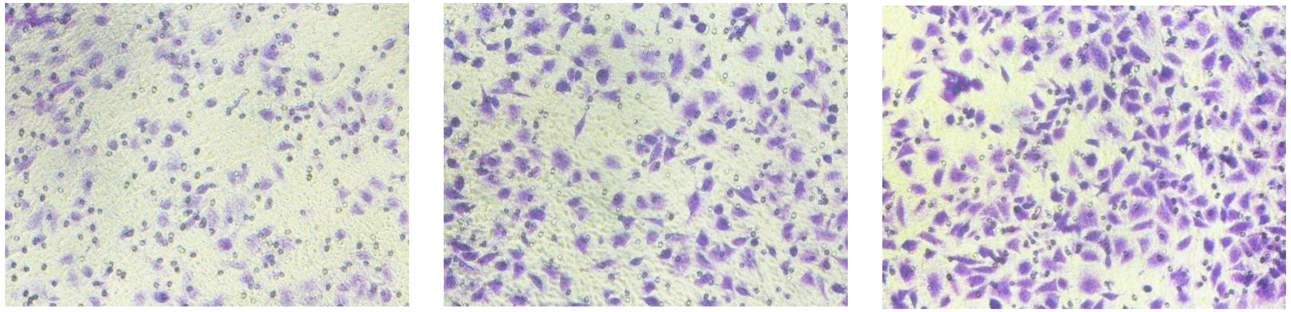

Figure3

Figure3B

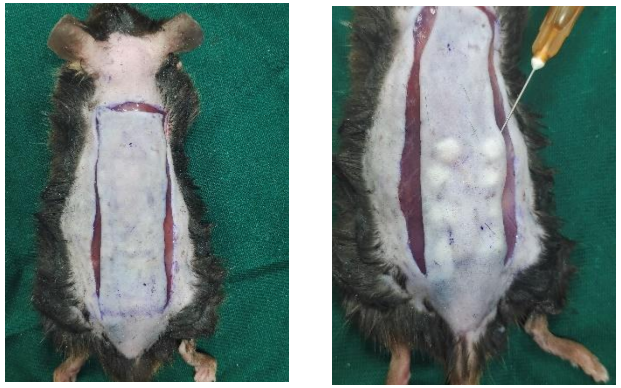

Figure3C

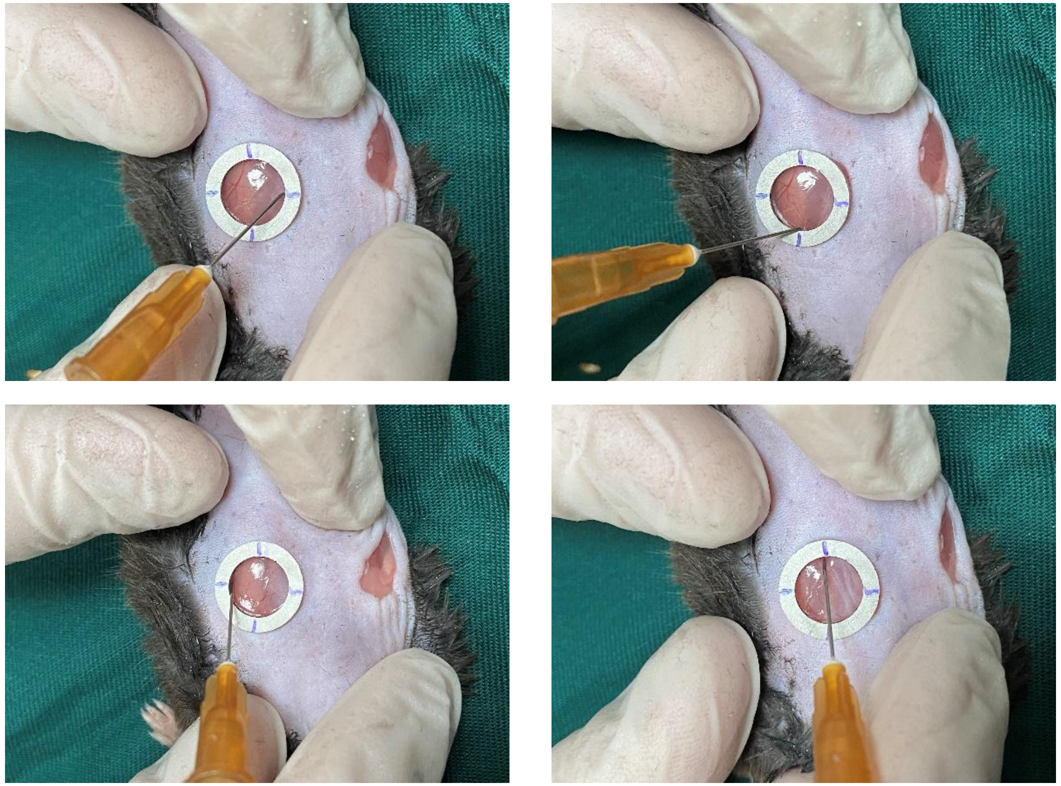

Figure4

Figure4A

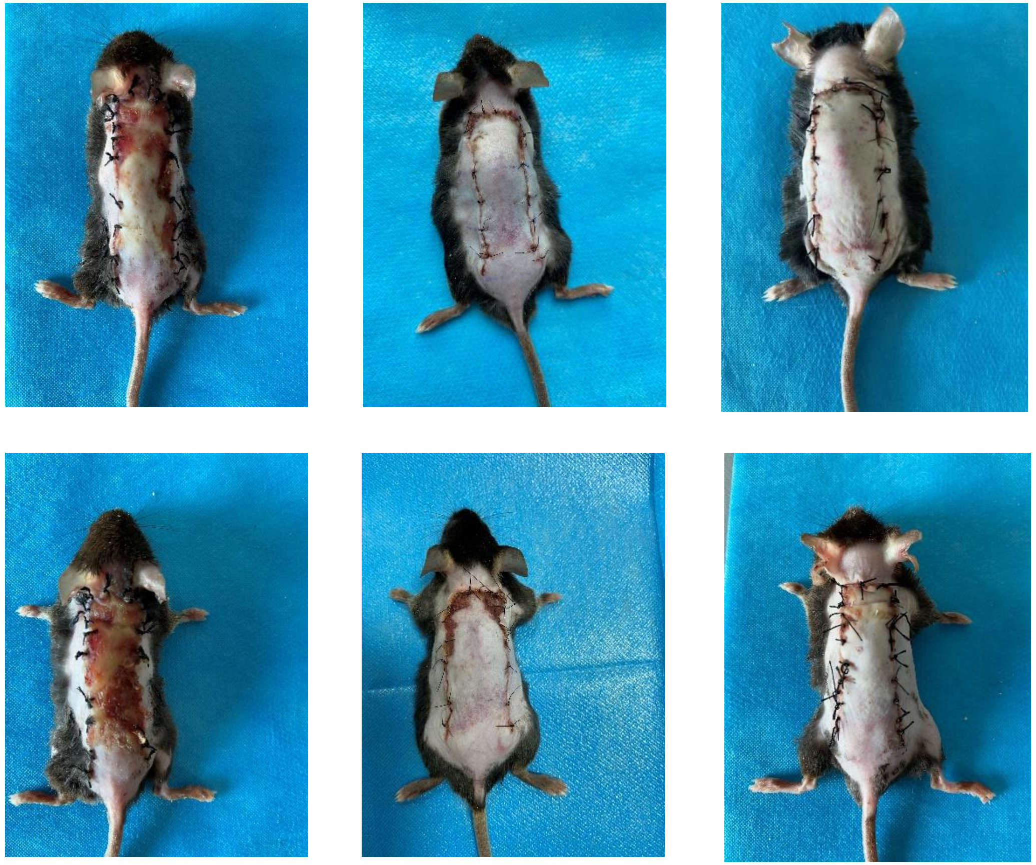

Figure4B

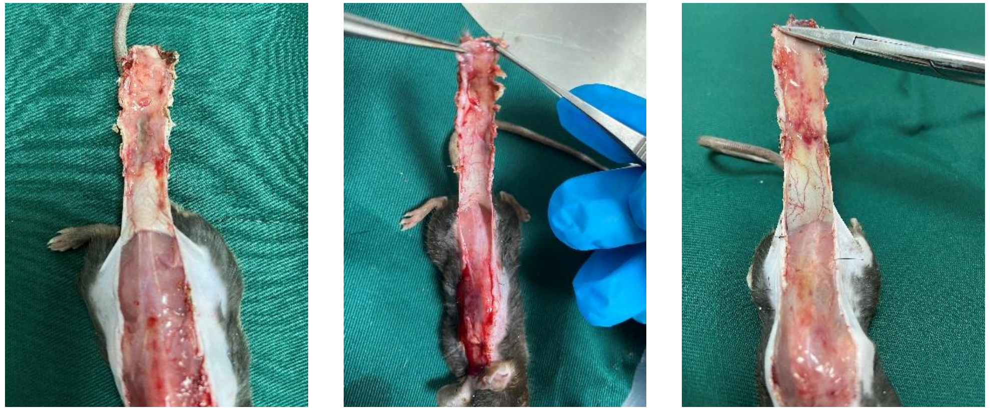

Figure4C

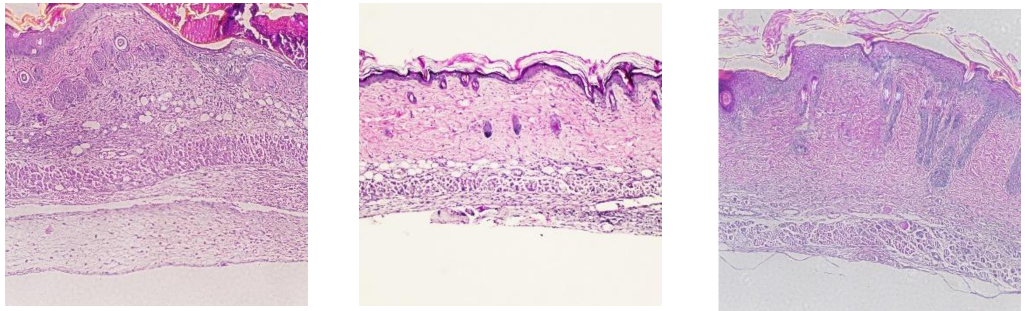

Figure4D

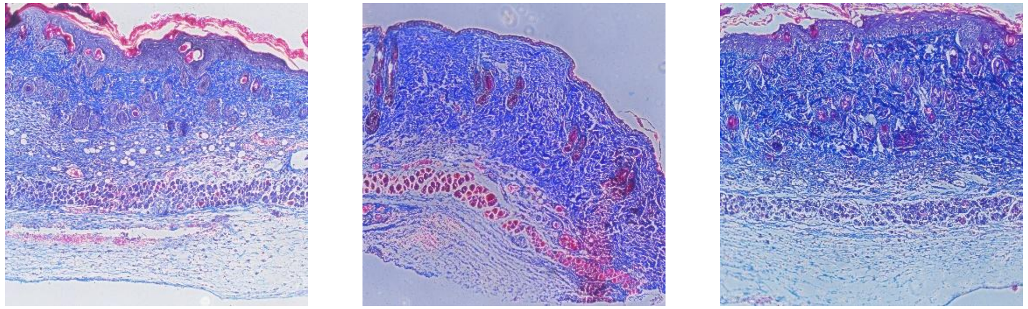

Figure5

Figure5A

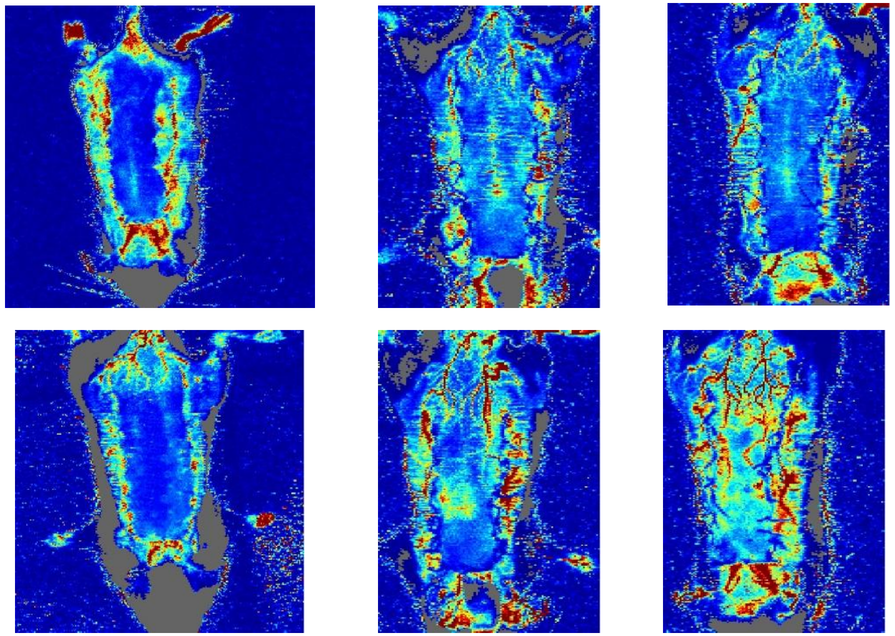

Figure5B

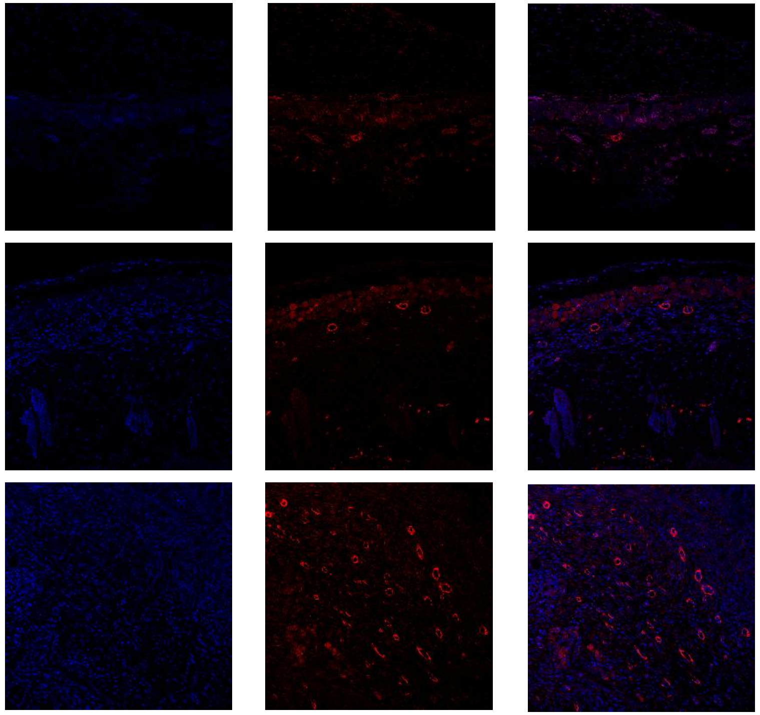

Figure5C

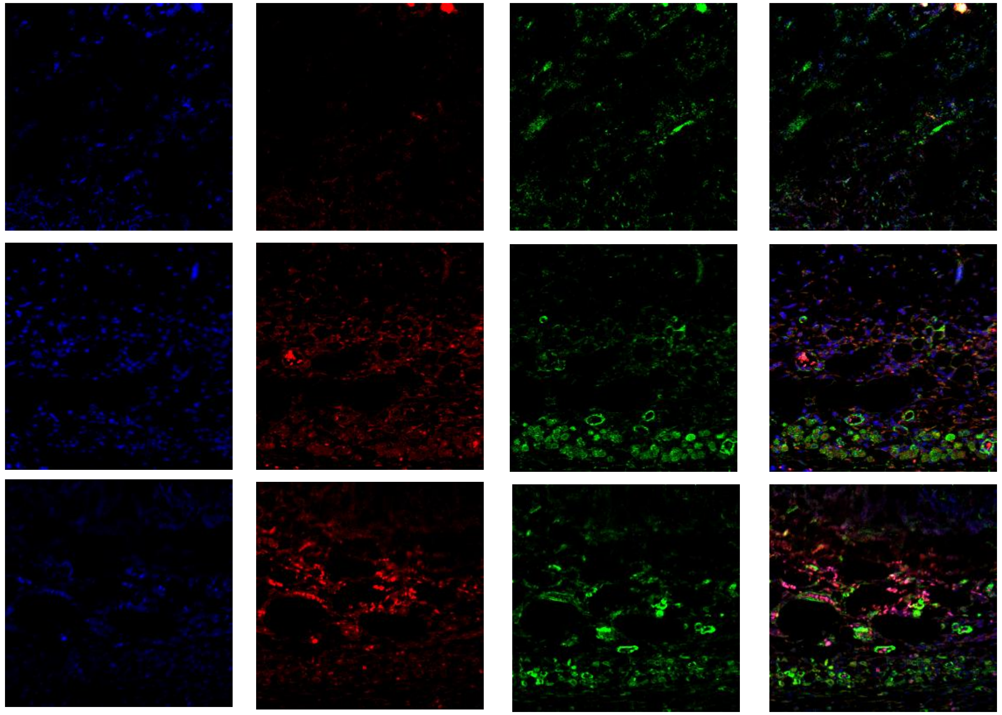

Figure6

Figure6A

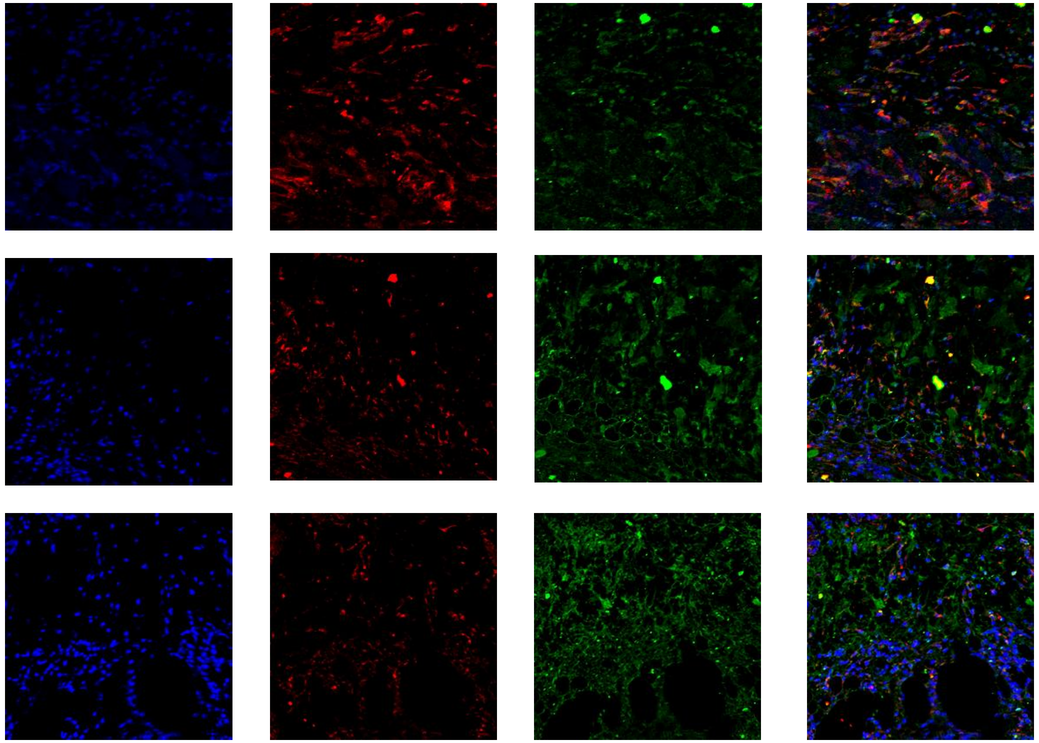

Figure7

Figure7A

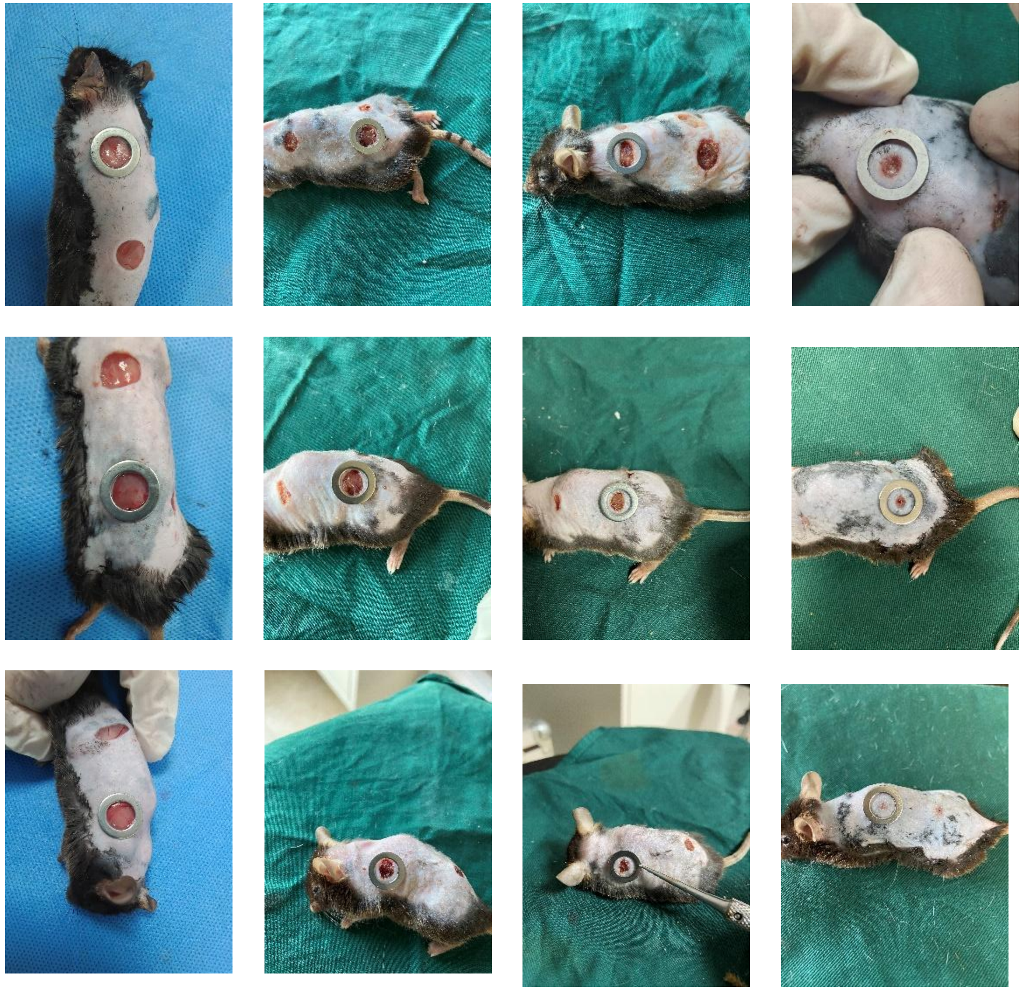

Figure7E

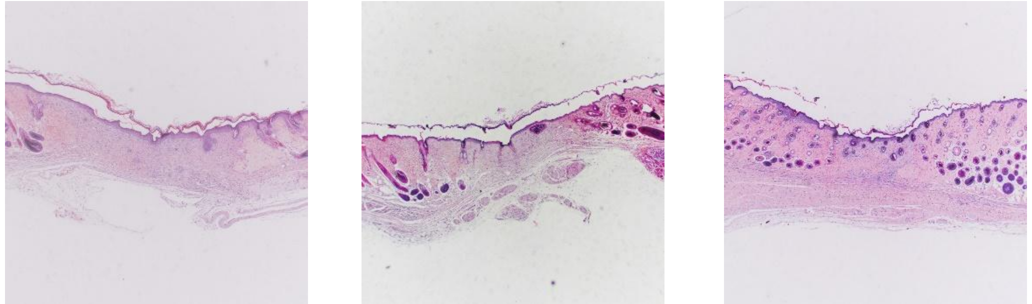

Figure8

Figure8A

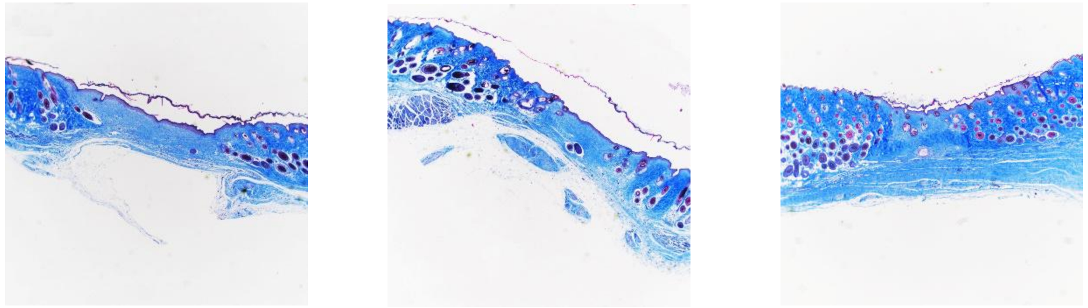

Figure8B

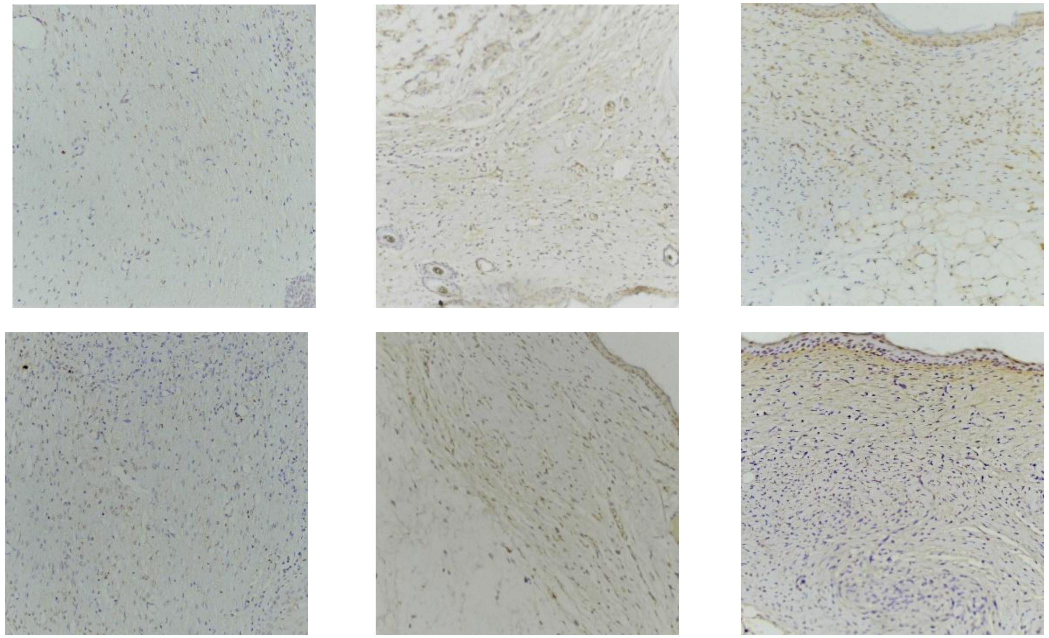

Figure8C

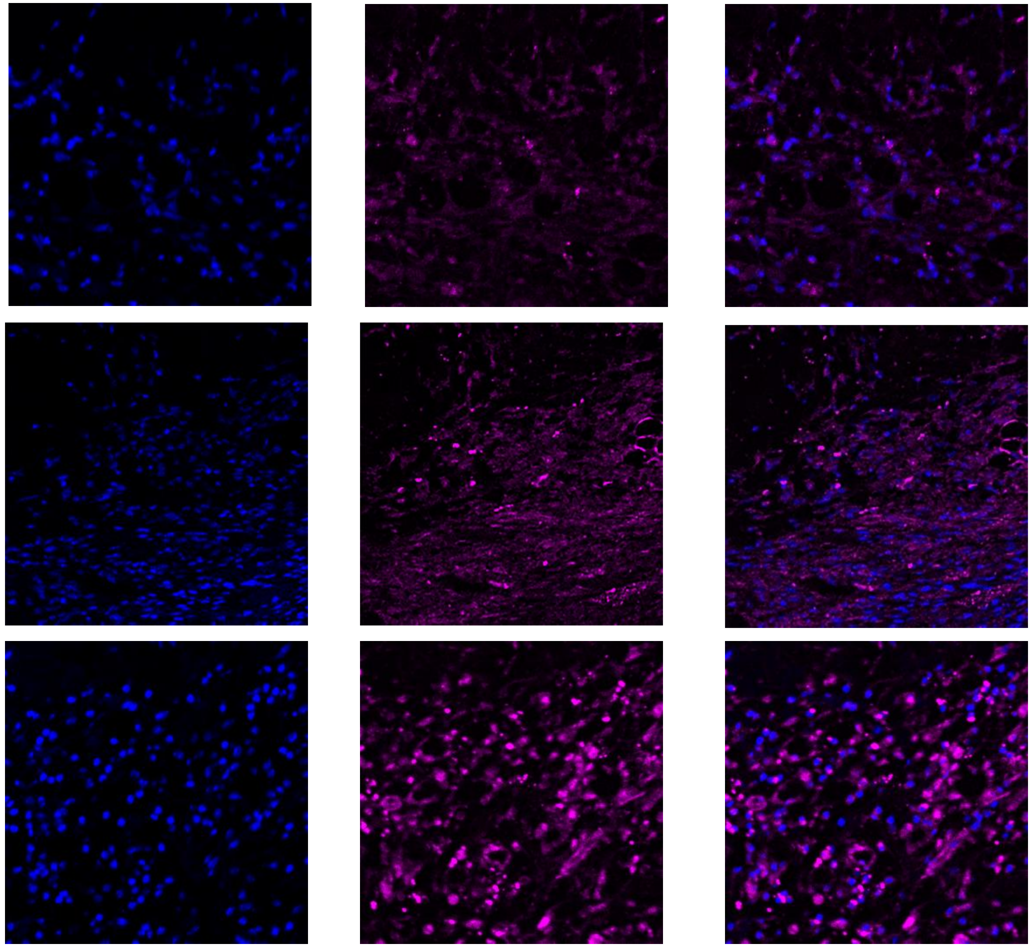

Figure9

Figure9A

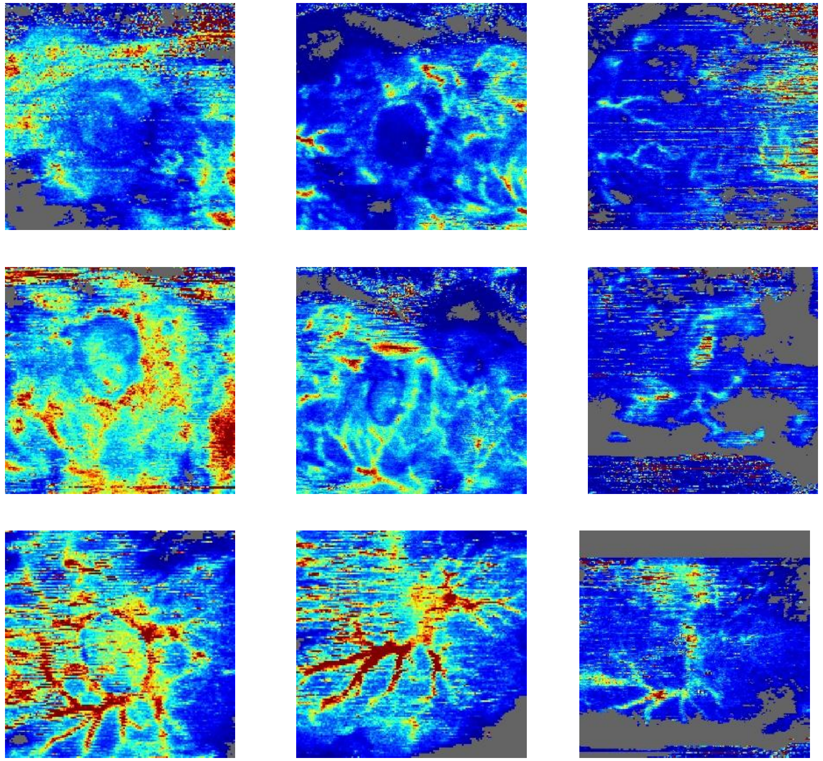

Figure9B

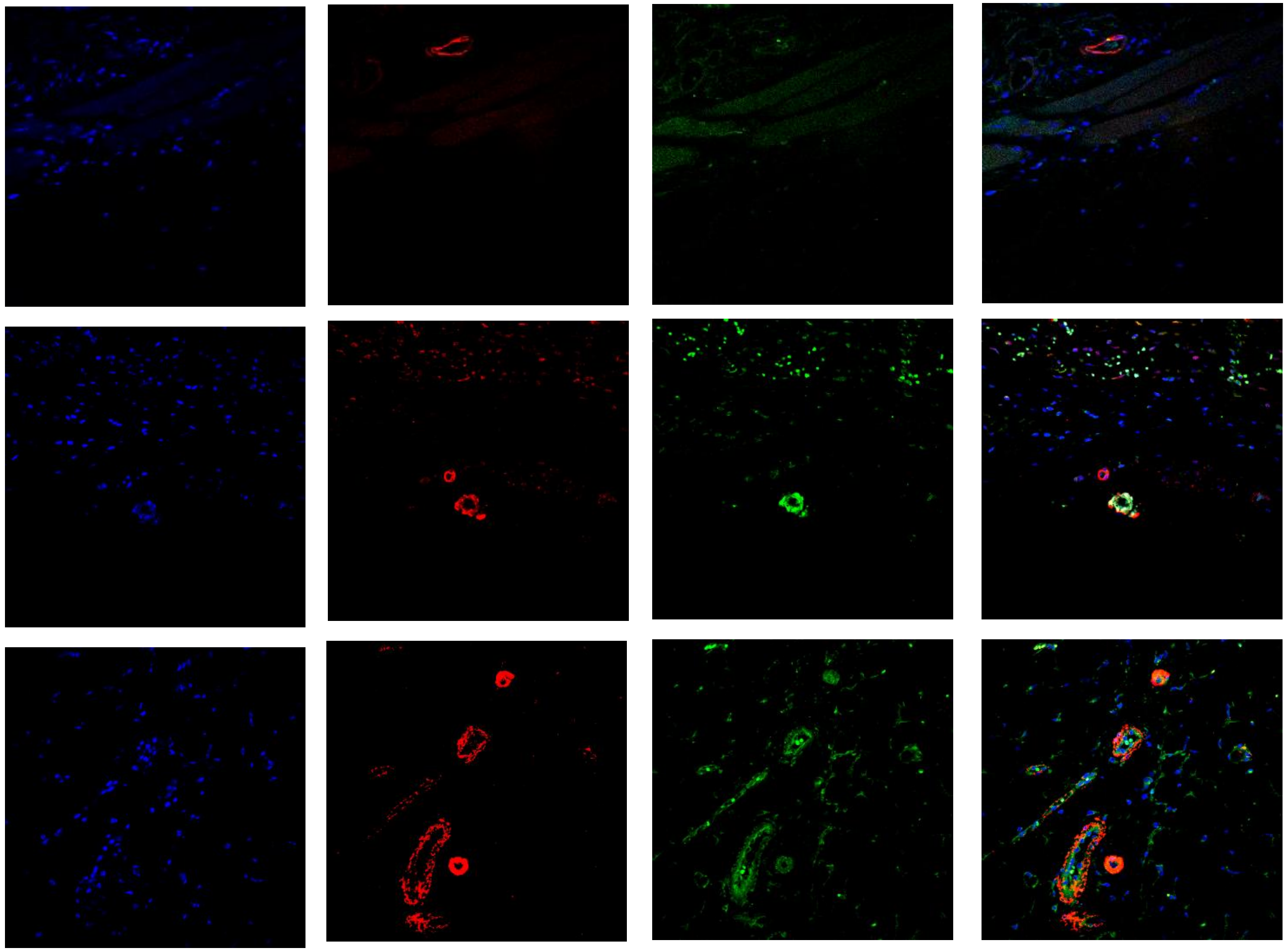

Figure9

Figure9C

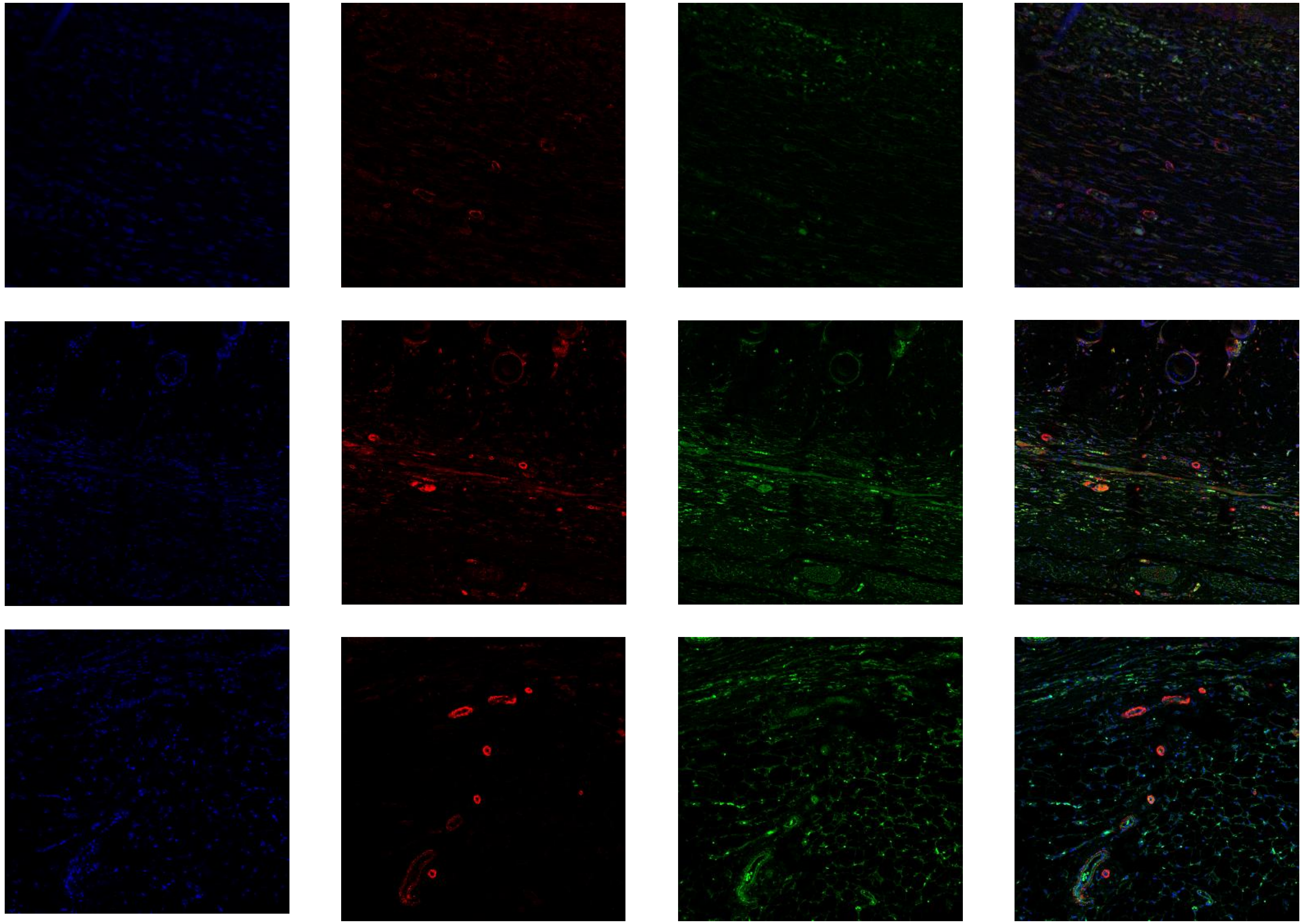

Figure10

Figure10A

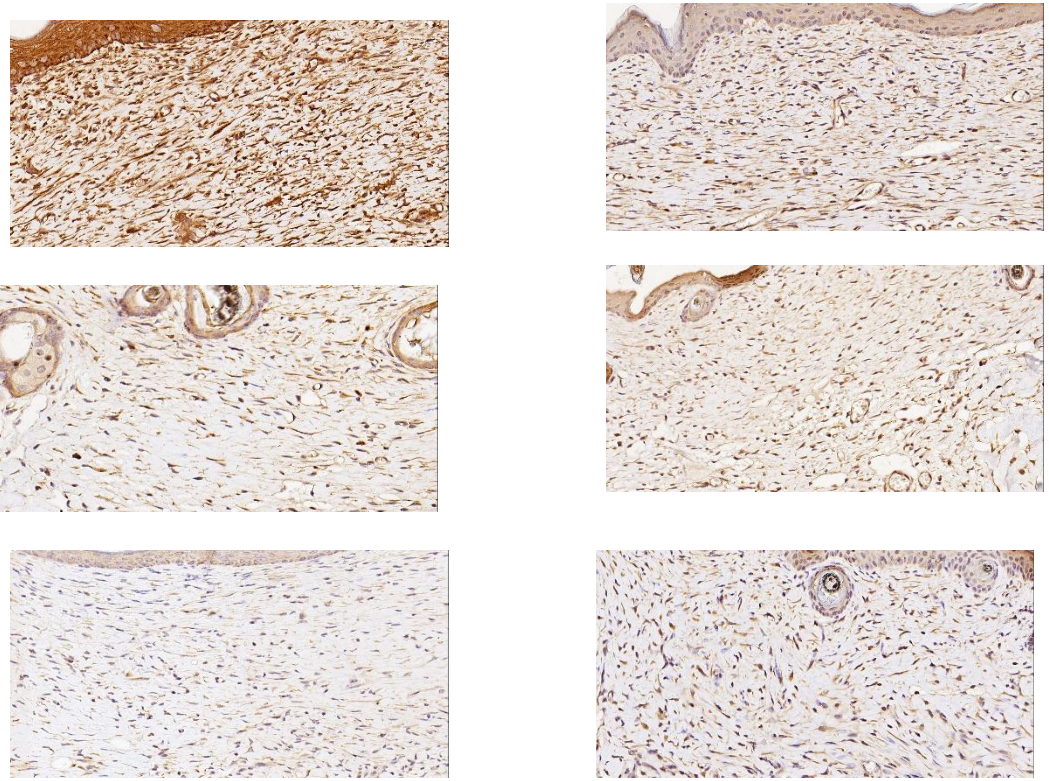

Figure10C

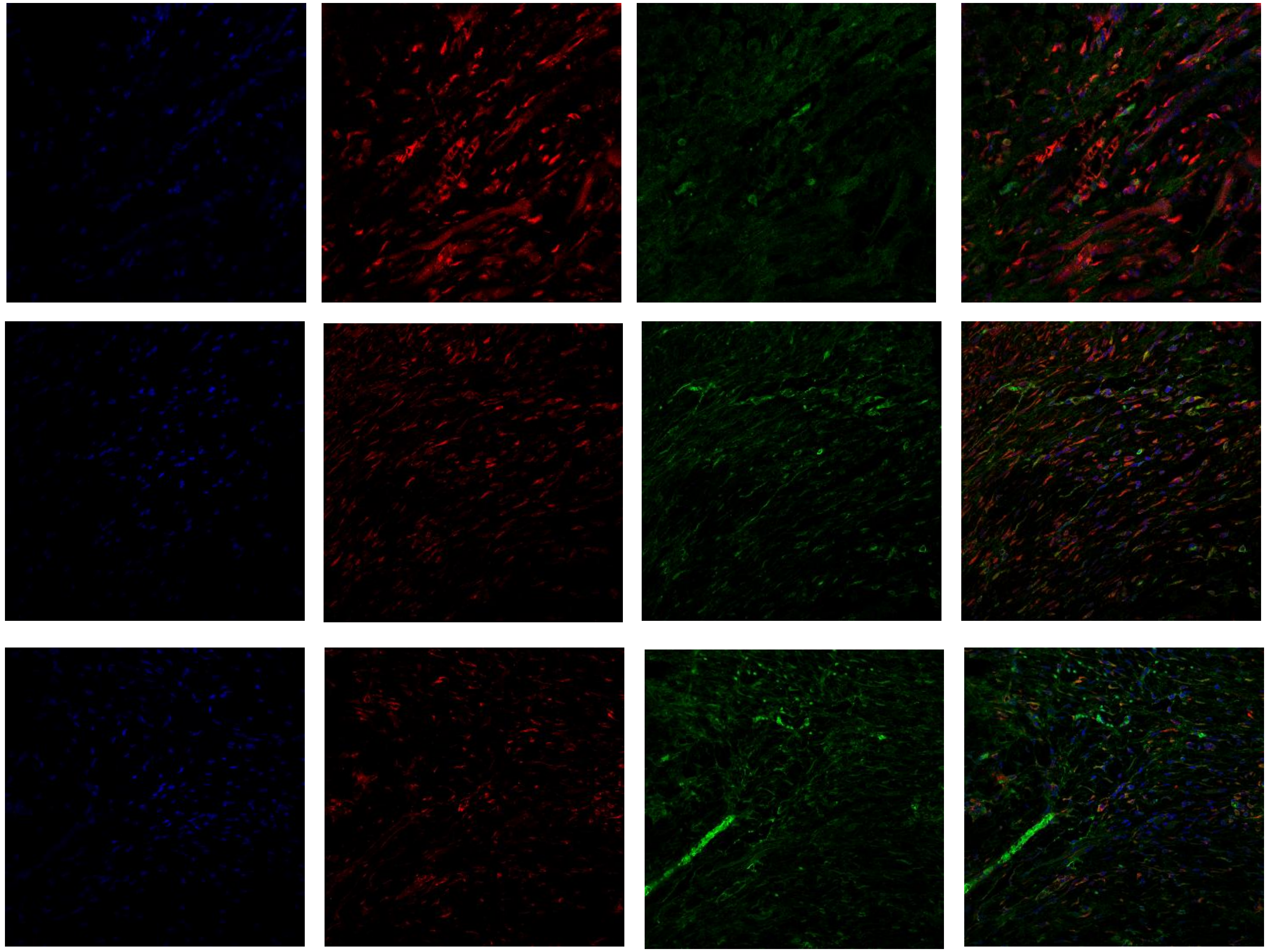

Figure10

Figure10E

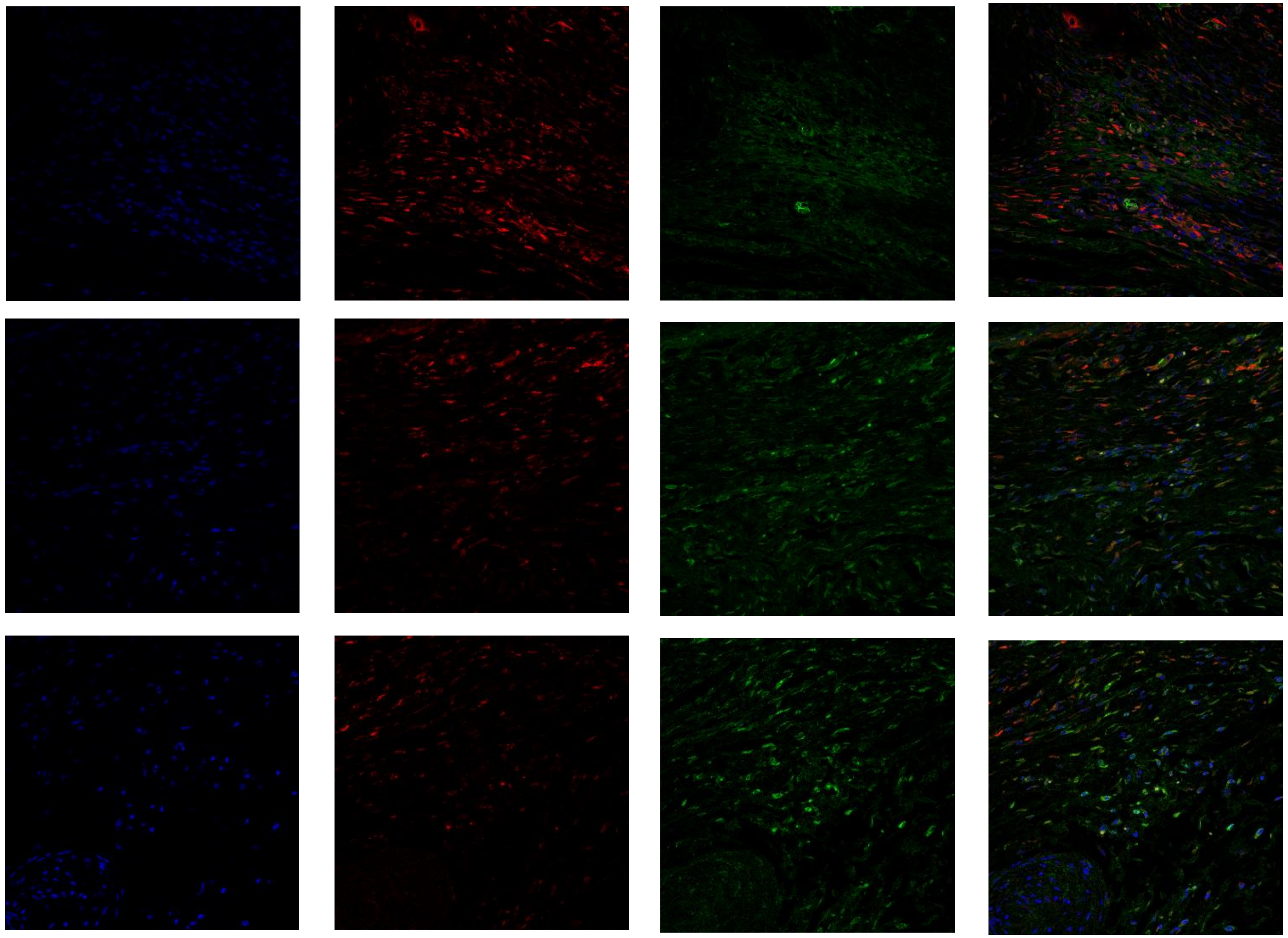

Figure11

Figure11A

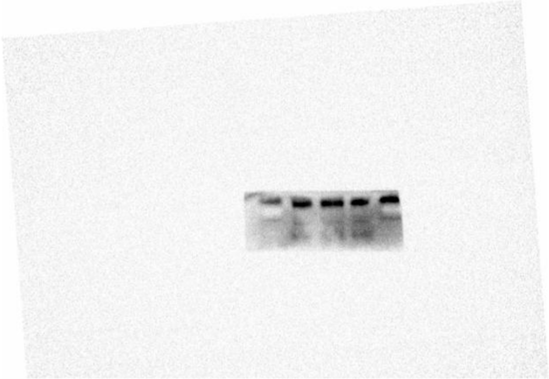

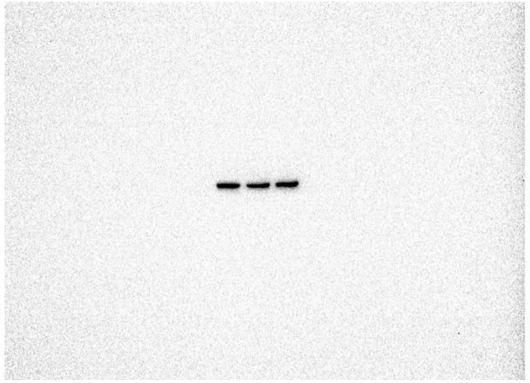

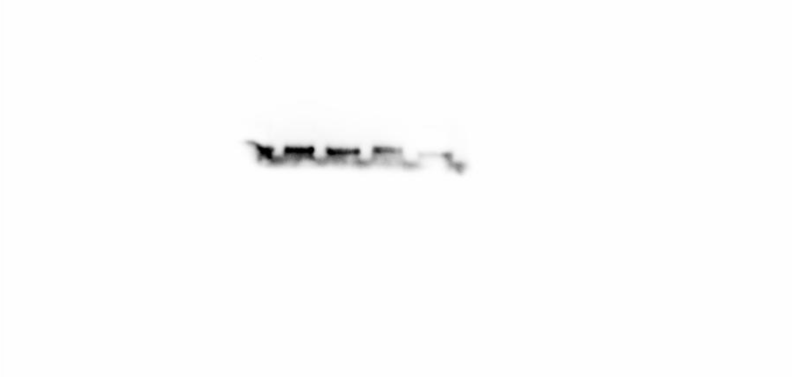

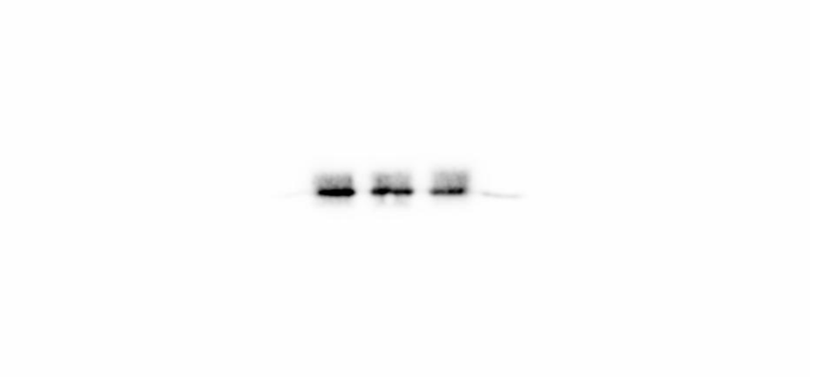

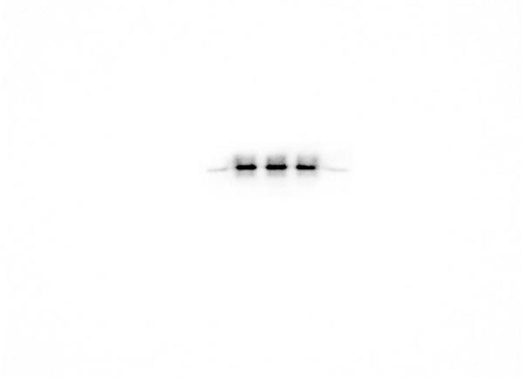

Supplement: Supplementary file 1 [file DataSheet1.PDF]
